# Analysis of integrated clinical trial protocols in early phases of medicinal product development

**DOI:** 10.1007/s00228-017-2335-y

**Published:** 2017-09-18

**Authors:** Kevin Fruhner, Gunther Hartmann, Thomas Sudhop

**Affiliations:** 1 Institute of Clinical Chemistry and Clinical Pharmacology, University Hospital, University of Bonn, Sigmund-Freud-Straße 25, 53127 Bonn, Germany; 2 Federal Institute for Drugs and Medical Devices (BfArM), Kurt-Georg-Kiesinger-Allee 3, 53175 Bonn, Germany

**Keywords:** Early-phase clinical trials, Integrated protocol, Non-integrated (standard) protocol, Substantial amendment, Federal Institute for Drugs and Medical Devices (BfArM)

## Abstract

**Purpose:**

While in the past, most clinical trial applications (CTAs) following non-integrated (standard) protocols were used to investigate one primary objective concerning a (new) drug, nowadays, the use of integrated protocols investigating multiple objectives within the same CTA becomes more and more popular. The aims of the present study were to investigate the usage and the impact of integrated protocols on regulatory activities and to find the motivation for their increasing use.

**Methods:**

Two thousand nine hundred sixty-nine phase I and I/II CTAs submitted to the German Federal Institute for Drugs and Medical Devices (BfArM) during the time period from August 1, 2004, until August 31, 2014, were analysed with regard to protocol and sponsor status, duration until initial authorisation and the number of substantial amendments and their respective approval times. Additionally, applicants who submitted integrated protocols to BfArM were interviewed with respect to their opinion on integrated protocols in an online survey.

**Results:**

The percentage of integrated protocols has constantly increased by approximately 10% within the last 10 years from 17.9% in 2004 to 28.2% in 2014. It could be shown that authorisation procedures with single integrated protocols take significantly longer until initial authorisation (58 vs. 53 days) requires more substantial amendments (1.9 vs. 1.2 amendments per CTA) and the approval of the entirety of amendments takes longer to process as compared to standard protocols (22 vs. 14 days). Nevertheless, applicants prefer the use of integrated protocols due to higher time and cost economy for the entire phase I development process.

**Conclusion:**

Although clinical trials (CTs) following integrated protocols are partly more time-consuming and costly, still, time and/or money may be saved during drug development due to the fact that overall, fewer CTs are needed than with standard protocols. Hence, the main reason for the increasing use of integrated protocols is improved time and cost efficiencies when conducting CTs.

**Electronic supplementary material:**

The online version of this article (10.1007/s00228-017-2335-y) contains supplementary material, which is available to authorized users.

## Introduction

In the development process and for marketing authorisation of medicinal products, clinical trials are essential. Their major goal is to test the efficacy and safety of (new) drugs. The drug development process consists of a series of clinical trials which is usually divided into four phases (I–IV) with different objectives [1–3]. While later-phase clinical trials are conducted in patients and focus on clinical efficacy and safety, most phase I trials are conducted in healthy subjects and focus primarily on human pharmacology and safety. The main aspects of these early trials are drug tolerability, pharmacokinetic and pharmacodynamic properties and interaction studies with food and other drugs. While pivotal phase III trials have a confirmatory approach in order to statistically prove clinical efficacy and safety, phase I trials are usually designed in a more exploratory manner. The exploratory nature of these trials allows the integration of several trial objectives without statistical disadvantages. Such clinical trial protocols in which two, three or even more primary objectives are analysed at the same time are called integrated protocols [4–6]. In such protocols, interdependent parts of a clinical trial are conducted consecutively. Examples for integrated protocols are clinical trials in which single ascending doses (SAD) as well as multiple ascending doses (MAD) with interaction studies and/or pharmacodynamic studies are combined [4, 5, 7–9]. With standard, non-integrated protocols, it would be necessary to conduct single clinical trials for each of these objectives.

With the implementation of the European Directive 2001/20/EC [10] into national law in 2004, both an approval from the competent federal higher authority and a positive opinion from the competent independent ethics committee became mandatory in Germany prior to the commencement of a clinical trial [11, 12]. In the past 10 years, the Federal Institute for Drugs and Medical Devices (BfArM), one of the two national competent authorities which authorise clinical trials in Germany, observed an increasing number of integrated trial protocols [13]. This mainly affects protocols of phase I and I/II clinical trials. Due to the complexity of integrated protocols, their handling by the competent authority proves to be more complicated and more time-consuming [5, 13–15] than the assessment of non-integrated phase I clinical trial protocols. Also, discussions and inquiries may occur more frequently. Nevertheless, the applicants can reduce the total number of submissions sent to the BfArM through the use of integrated protocols. This raised the question of whether time and financial expenses could be saved by using integrated protocols in the authorisation process of clinical trials. The aim of the present study was therefore to analyse how many integrated phase I and I/II protocols are actually being evaluated by the BfArM and whether the utilisation of integrated protocols can result in time and cost savings in the development process and especially in the clinical trial authorisation process. In order to analyse the perspective and motivation of the applicants, we additionally conducted an anonymous online survey. In this survey, applicants were interviewed as to their opinions and experiences related to integrated protocols in phase I and I/II clinical trials.

## Methods

### Database analysis

#### Data sources and data set selection

**Fig. 1:**
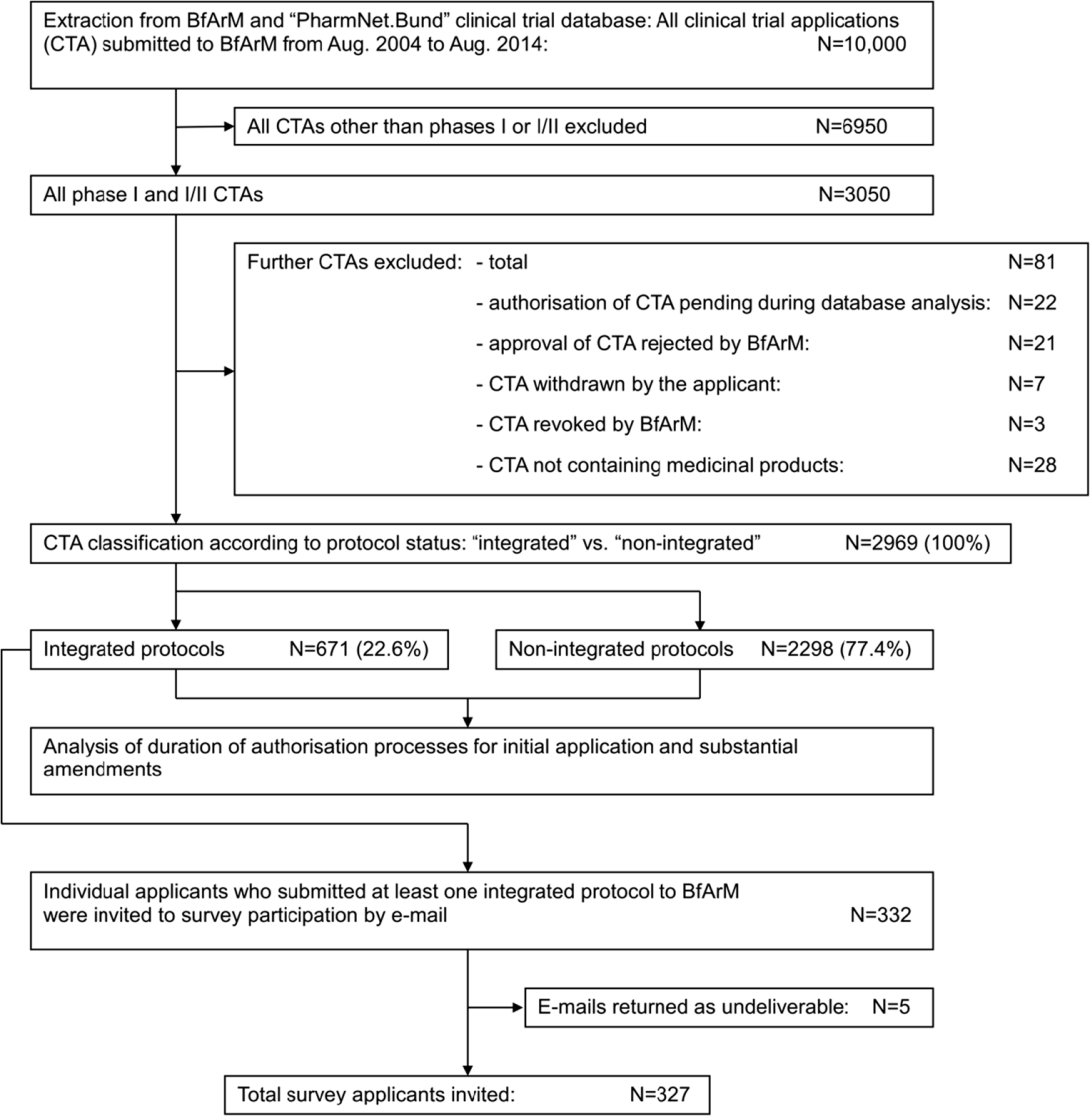
Data set selection and survey participant selection. All clinical trial applications (CTA) submitted to the Federal Institute for Drugs and Medical Devices (BfArM) between August 2004 and August 2014 (*N* = 10,000) were collected from the PharmNet.Bund database as well as from the in-house archive of the BfArM. After exclusion of all CTAs other than phase I and I/II (*N* = 6950), further CTAs were removed from the data set (*N* = 81). In this regard, CTAs where the initial authorisation was still pending when the database analysis was performed (*N* = 22), CTAs that the BfArM rejected (*N* = 21), CTAs that were withdrawn by the applicant (*N* = 7), CTAs that were revoked by the BfArM (*N* = 3) and CTAs that were not associated with medicinal products (*N* = 28) were excluded from the analysis. The remaining phase I and I/II CTAs (*N* = 2969) were classified according to their protocol status. *N* = 671 met the criteria for an integrated protocol, and *N* = 2298 were identified as non-integrated protocols. Applicants who submitted at least one integrated protocol to BfArM were invited by e-mail to participate in the online survey (*N* = 332). As five e-mails were returned as undeliverable, a total of *N* = 327 applicants were invited to participate in the survey

In order to ensure an appropriate sample size, the first 10,000 clinical trial applications submitted to the BfArM which were reviewed under the scope of Directive 2001/20/EC were selected. The applications were submitted between August 1, 2004, and August 31, 2014. Primary data source was the national ‘PharmNet.Bund’ database [16, 17] which is linked to the EUDRA-CT database of the European Medicines Agency (EMA). The EUDRA-CT database contains all clinical trial applications in the European Union since 2004 which were submitted under the scope of the European clinical trial legislation [18]. Additionally, workflow data were merged from the internal workflow database of the BfArM. Of the 10,000 applications, all phase II, III and IV clinical trial protocols were excluded from the analysis. Furthermore, clinical trial applications which were rejected or withdrawn during the authorisation review were excluded from the analysis. After exclusion of all irrelevant protocols (s.a.), 2969 phase I and I/II clinical trial protocols remained in the analysis (Fig. 1).

#### Variables

The data set of 2969 clinical trial applications was classified according to protocol type (‘integrated’ vs. ‘non-integrated’), sponsor status (‘commercial’ vs. ‘non-commercial’) and duration of the authorisation process. For the latter, the time period from the initial application up to the final approval was calculated. In order to evaluate subsequent regulatory activities, the number of submitted substantial amendments as well as their overall time to approval was also calculated. Additionally, integrated protocols were classified into subgroups according to their primary and secondary objectives and endpoints.

### Questionnaire regarding integrated protocols

All applicants who submitted at least one integrated phase I or I/II trial protocol from August 1, 2004, to August 31, 2014 were invited to participate in an online survey concerning their opinion and experience on integrated trial protocols. The online survey was conducted with the open-source software product ‘LimeSurvey’ version 2.05+ build 150310 [19]. For this purpose, the programme was installed on the in-house servers of the BfArM. To ensure that every applicant could complete only one questionnaire, individualised invitation links were sent by the survey programme. The system was configured in such a manner that the returning answers were completely anonymised and no inferences with regard to the identity of the respondent could be drawn [20]. The questionnaire consisted of 21 questions, divided into three main categories regarding ‘motivation for the utilisation of integrated protocols’, ‘experience with integrated protocols’ and ‘expenditure for the execution of integrated protocols’. The questionnaire mostly used multiple-choice questions with six possible answers. Partially completed questionnaires were also included in the analysis provided that at least one item had been answered. In order to increase the return rate, reminder e-mails were sent to the invited applicants.

#### Measurements

Subgroup analyses were performed with respect to different responder types. Results were compared according to the experience of the applicants with integrated protocols (‘≤ 30%’ vs. ‘> 30% integrated protocols in the past five years’) as well as the planned future use of integrated protocols (‘yes’ vs. ‘no’ or ‘do not know’). Another subgroup analysis compared the responders based on their opinion on the duration of the execution of a clinical trial following an integrated protocol compared to a standard, non-integrated protocol (‘shorter’ vs. ‘longer’). Two further subgroup analyses compared the survey participants based on their experience regarding substantial amendments and major problems when dealing with integrated protocols (‘more’, ‘less’ or ‘same amount’). Additionally, the responders were compared according to their opinion on whether the use of integrated protocols can save time as well as money in comparison with non-integrated protocols (‘yes’ vs. ‘no’). Finally, the last subgroup analysis differentiated the participants of the online survey with reference to their sponsor status (‘commercial’ vs. ‘non-commercial’). Since the response rate for some questions with tendency options was rather low, the options ‘totally agree’, ‘agree’ and ‘rather agree’ were clustered as ‘agreement’; correspondingly, the answers ‘totally disagree’, ‘disagree’ and ‘rather disagree’ were summarised as ‘disagreement’.

#### Statistical methods

The statistical analysis was performed using the IBM© SPSS© statistic software package version 21 for Microsoft Windows operating systems. Exploratory parameters mean, median, standard deviation, spread, minimum and maximum were calculated for each parameter. For normally distributed data, the two-sided *t* test was performed; for all others, the non-parametrical Mann-Whitney *U* test was employed. Time-dependent correlations were assessed by Pearson’s correlation coefficient. For the assessment of the questionnaire, the chi-square test and the Fisher’s exact test were used.

## Results

### Database analysis

Six hundred seventy-one (22.6%) of the 2969 analysed phase I and I/II clinical trial protocols met the criteria for integrated protocols. Two thousand two hundred ninety-eight (77.4%) did not meet these criteria and were therefore assigned as non-integrated protocols. Eighty-one phase I and I/II protocols were excluded prior to the analysis because of various reasons which are depicted in Fig. 1.

**Fig. 2:**
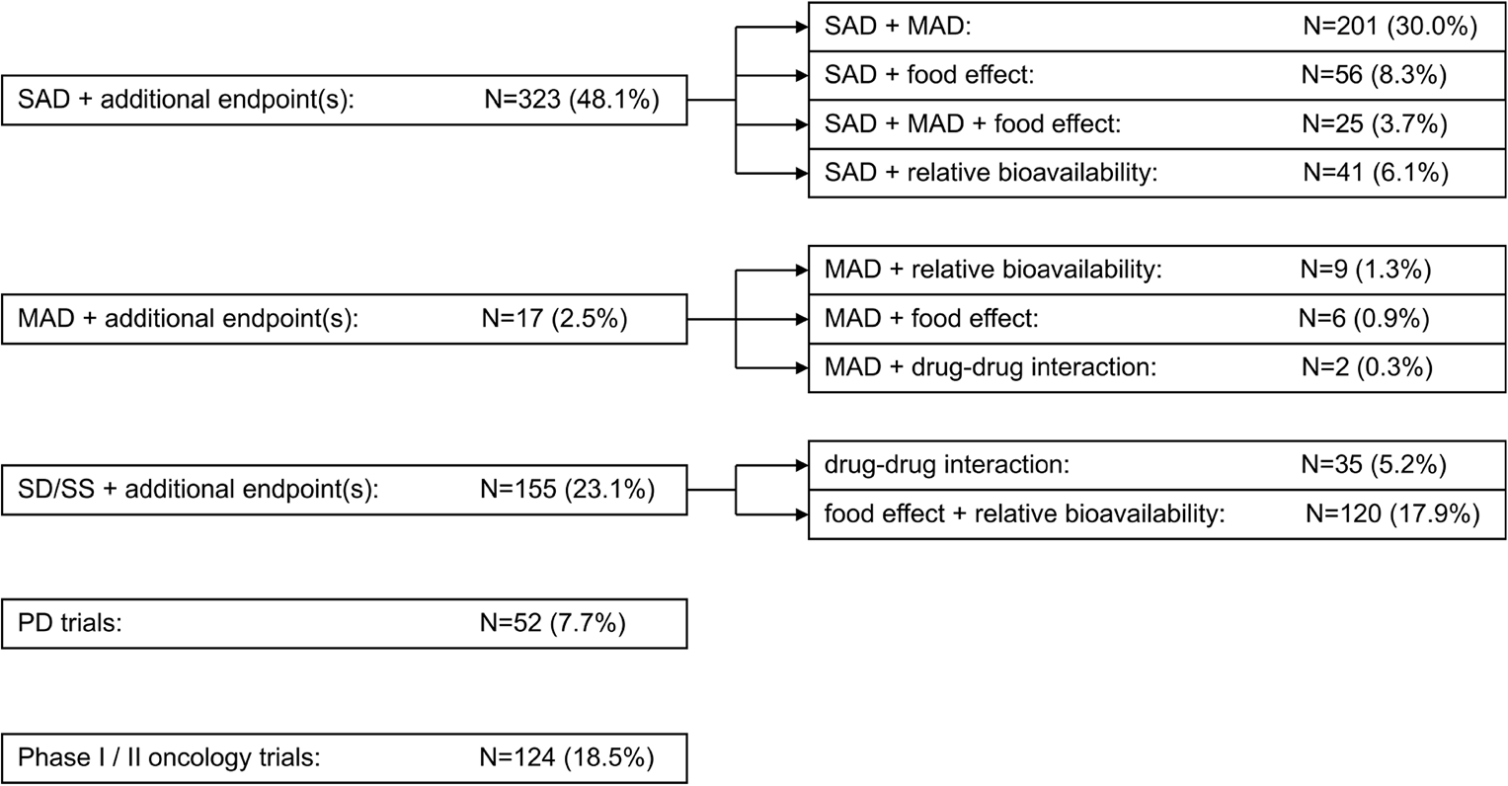
Classification of integrated protocols. According to their primary and secondary endpoints, the integrated protocols were classified into five major subgroups ‘SAD + additional endpoint(s)’, ‘MAD + additional endpoint(s)’, ‘SD/SS + additional endpoint(s)’, ‘PD trials’ and ‘Phase I/II oncology trials’. These subgroups partially consisted of further smaller subgroups which are displayed as supplemental information only. The total numbers as well as the percentage values are displayed. SAD single ascending dose trials, MAD multiple ascending dose trials, SD/SS trials using stable dose(s) (single or multiple) or steady-state conditions, PD trials with primary pharmacodynamic objectives

The analysis of the primary and secondary objectives and endpoints of the integrated trial protocols revealed five major subgroups: SAD trials with additional endpoints, MAD trials with additional endpoints, trials using stable dose(s) (single or multiple) or steady-state conditions (SD/SS) with additional endpoints, pharmacodynamic trials (PD) with additional endpoints and phase I/II oncology trials. These subgroups partially consisted of further smaller subgroups which are displayed as supplemental information only in Fig. 2.

**Fig. 3:**
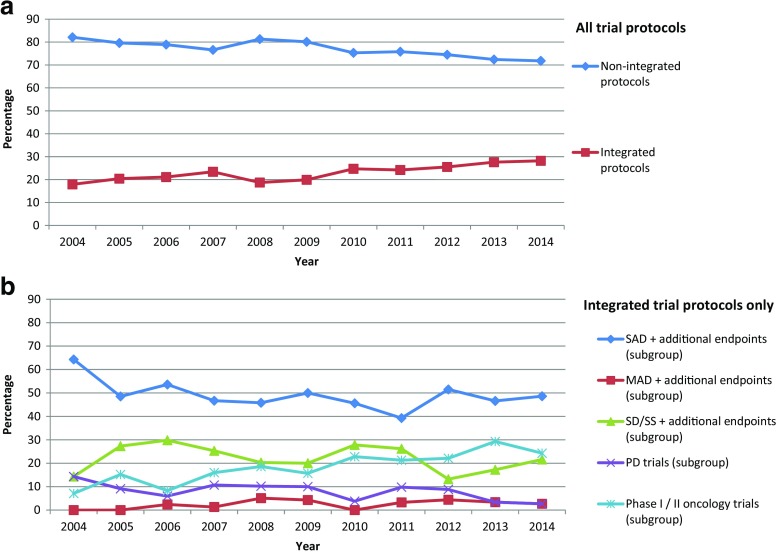
Integrated protocols over time. **a** Percentage of integrated protocols (*N* = 671) in comparison to standard protocols (*N* = 2298) for the years 2004 to 2014. **b** Percentage of the five major subgroups of integrated protocols (‘SAD + additional endpoint(s)’, ‘MAD + additional endpoint(s)’, ‘SD/SS + additional endpoint(s)’, ‘PD trials’ and ‘Phase I/II oncology trials’) for the years 2004 to 2014 (*N* = 671). SAD single ascending dose trials, MAD multiple ascending dose trials, SD/SS trials using stable dose(s) (single or multiple) or steady-state conditions, PD trials with primary pharmacodynamic objectives

A detailed review of the proportion of integrated protocols in phase I and I/II trials revealed a more or less steady increase over the decade since 2004. While in 2004, merely 17.9% of all phase I and I/II clinical trial protocols authorised by the BfArM were integrated, the percentage increased to 20.4% in 2005, hit the 25% mark in 2012 and reached a maximum percentage of 28.2% in 2014 (Fig. 3a). When analysing the trial subgroups, it became obvious that most integrated protocols were based on SAD trials with additional endpoints, followed by SD/SS trials with additional endpoints and phase I/II oncology trials. However, there was no clear trend towards emergence or abolition of one specific subgroup detectable over the decade (Fig. 3b).

When analysing the sponsor status, approximately 90% of the 2969 clinical trial applications were submitted by commercial sponsors (*N* = 2660; 89.6%) either by pharmaceutical companies directly or indirectly by contract research organisations (CROs). Only 10.4% (*N* = 309) were sponsored by non-commercial sponsors, primarily by university hospitals. However, both commercial and non-commercial sponsors used integrated protocols almost to the same proportion (22.6 vs. 22.3%).

**Fig. 4:**
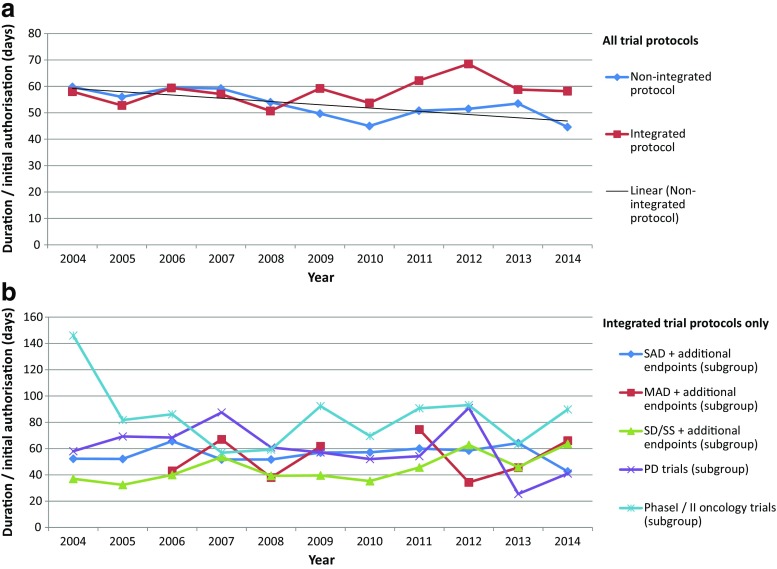
Duration until initial authorisation over time. **a** Duration until initial authorisation of a clinical trial application displayed in days for integrated protocols (*N* = 671) in comparison to non-integrated protocols (*N* = 2298) for the years 2004 to 2014. Correlation of duration over the time for non-integrated protocols: *r* = − 0.76 (*p* = 0.007), for integrated protocols: not significant. **b** Duration until initial authorisation of an integrated trial application displayed for the five major subgroups of integrated protocols (‘SAD + additional endpoint(s)’, ‘MAD + additional endpoint(s)’, ‘SD/SS + additional endpoint(s)’, ‘PD trials’ and ‘Phase I/II oncology trials) for the years 2004 to 2014 (*N* = 671). SAD single ascending dose trials, MAD multiple ascending dose trials, SD/SS trials using stable dose(s) (single or multiple) or steady-state conditions, PD trials with primary pharmacodynamic objectives

It could be shown that the time from submission until initial authorisation of a clinical trial application differs significantly between both types of clinical trial protocols. The duration from submission until initial authorisation of an integrated clinical trial protocol averages about 58 days, whereas non-integrated protocols were approved within 53 days after submission (*p* < 0.001, Table 1). When analysing the duration until initial authorisation of a clinical trial application (CTA) over the last 10 years, it became obvious that there was a significant trend towards shorter approval times for non-integrated protocols (*r* = − 0.76; *p* = 0.007, Fig. 4a). In contrast to that, there was a non-significant trend towards increasing approval times for integrated protocols over the decade. The most prominent subgroup (SAD trials) showed more or less stable approval times over time (Fig. 4b).

**Table 1:** Duration/initial authorisation

	Valid values	Missing values	Duration/initial authorisation (days)		Standard deviation
			Mean	Median	
Total data set	2921 (98.4%)	48 (1.6%)	54.4	46	33.1
Non-integrated protocols	2261 (76.2%)	37 (1.2%)	53.3	45	33.7
Integrated protocols	660 (22.2%)	11 (0.4%)	58.0*	51	31.1
SAD + additional endpoint(s)	320 (47.7%)^a^	3 (0.4%)	56.8	52	27.4
MAD + additional endpoint(s)	17 (2.5%)^a^	0 (0.0%)	50.7	49	18.9
SD/SS + additional endpoint(s)	152 (22.7%)^a^	3 (0.4%)	43.3	35	21.9
PD trials	52 (7.7%)^a^	0 (0.0%)	66.5	63	32.5
Phase I/II oncology trial	119 (17.7%)^a^	5 (0.7%)	77.6	78	39.1
Commercial sponsors	2624 (88.4%)	36 (1.2%)	51.5**	45	28.6
Non-commercial sponsors	297 (10.0%)	12 (0.4%)	80.3	64	53.4

The average duration until initial authorisation of a clinical trial application varies depending on the protocol type (integrated/non-integrated) and on the sponsor status (commercial/non-commercial). Both the differences between integrated and non-integrated protocols as well as the differences between commercial and non-commercial sponsors were significant. Furthermore, the average duration until initial authorisation of an integrated trial protocol varies significantly depending on the primary and secondary endpoints of the trial

*SAD* single ascending dose trials, *MAD* multiple ascending dose trials, *SD/SS* trials using stable dose(s) (single or multiple) or steady-state conditions, *PD* trials with primary pharmacodynamic objectives

*p < 0.001 (integrated protocols compared to non-integrated); **p < 0.001 (commercial sponsors compared to non-commercial)

^a^Percentage refers to integrated protocols only

Furthermore, a significant difference in the time from submission until initial authorisation between commercial and non-commercial sponsors was observed. While phase I and I/II protocols submitted by commercial sponsors could be approved within 52 days on average, clinical trial applications submitted by non-commercial sponsors required a mean handling time of 80 days (*p* < 0.001, Table 1).

The BfArM assessment process of a CTA results either in a direct approval or in one or more deficiency letters with respect to validation issues and/or other regulatory issues which lead to an initial non-acceptance of the CTA. One determining factor that has a huge impact on the varying time periods from submission until authorisation is the time the applicant needs for revision in case of deficiency letters. One thousand four hundred twenty (47.8%) of the analysed CTAs were initially not accepted by the BfArM due to content deficiency issues. It could be shown that the initial non-acceptance of a CTA significantly depends on the type of protocol. While 56% (373) of the integrated protocols exhibited content deficiency issues, these were present in only 46% (1047) of the non-integrated protocols (*p* < 0.001). No significant differences of initial non-acceptance in relation to the sponsor status (commercial vs. non-commercial) could be found.

In order to investigate the impact of the protocol type on subsequent regulatory activities after the initial approval, the average number of submitted substantial amendments per clinical trial as well as the duration for their approval process was analysed. While an average of 1.9 amendments were submitted for CTAs with integrated protocols, applicants of clinical trials following non-integrated protocols submitted only 1.2 substantial amendments per trial (*p* < 0.001, Table 2).

**Table 2:** Number/substantial amendments per clinical trial application

	Valid values	Missing values	Number/substantial amendments (per CTA)		Standard deviation
			Mean	Median	
Total data set	2969 (100%)	0	1.4	1	2.3
Non-integrated protocols	2298 (77.4%)	0	1.2	0	2.1
Integrated protocols	671 (22.6%)	0	1.9*	1	2.8
SAD + additional endpoint(s)	323 (48.1%)^a^	0	1.6	1	2.2
MAD + additional endpoint(s)	17 (2.5%)^a^	0	1.9	2	2.1
SD/SS + additional endpoint(s)	155 (23.1%)^a^	0	0.8	0	1.1
PD trials	52 (7.7%)^a^	0	2.2	1	2.7
Phase I/II oncology trials	124 (18.5%) ^a^	0	4.1	4	4.1
Commercial sponsors	2660 (89.6%)	0	1.4	1	2.3
Non-commercial sponsors	309 (10.4%)	0	1.4	1	2.0

The average number of substantial amendments per clinical trial application varies depending on the protocol type (integrated/non-integrated) and on the sponsor status (commercial/non-commercial). While significant differences between integrated and non-integrated protocols could be found, no significant differences between commercial and non-commercial sponsors were detected. Furthermore, the average number of substantial amendments per integrated trial protocol varies significantly depending on the primary and secondary endpoints of the trial

*SAD* single ascending dose trials, *MAD* multiple ascending dose trials, *SD/SS* trials using stable dose(s) (single or multiple) or steady-state conditions, *PD* trials with primary pharmacodynamic objectives

*p < 0.001 (integrated protocols compared to non-integrated)

^a^Percentage refers to integrated protocols only

The total duration for the approval of all substantial amendments of a CTA lasted an average of 22 days for integrated protocols which was significantly longer compared to 14 days for non-integrated trial protocols (*p* < 0.001, Table 3). No significant differences between numbers and processing time of substantial amendments in relation to the sponsor status (commercial vs. non-commercial) could be detected (Tables 2 and 3).

**Table 3:** Duration/substantial amendments

	Valid values	Missing values	Duration/substantial amendments (days)		Standard deviation
			Mean	Median	
Total data set	2771 (93.3%)	198 (6.7%)	16.0	0	30.8
Non-integrated protocols	2162 (72.8%)	136 (4.6%)	14.2	0	28.2
Integrated protocols	609 (20.5%)	62 (2.1%)	22.3*	10	38.2
SAD + additional endpoint(s)	297 (44.3%)^a^	26 (3.9%)	19.4	12	30.8
MAD + additional endpoint(s)	16 (2.4%)^a^	1 (0.1%)	21.2	14	31.9
SD/SS + additional endpoint(s)	151 (22.5%)^a^	4 (0.6%)	8.4	0	12.7
PD trials	46 (6.9%)^a^	6 (0.9%)	23.5	14	28.9
Phase I/II oncology trials	99 (14.8%)^a^	25 (3.7%)	52.2	38	64.7
Commercial sponsors	2491 (83.9%)	169 (5.7%)	16.1	0	31.5
Non-commercial sponsors	280 (9.4%)	29 (0.9%)	15.0	0	24.6

The average duration for the approval of all substantial amendments of a CTA varies depending on the protocol type (integrated/non-integrated) and on the sponsor status (commercial/non-commercial). While significant differences between integrated and non-integrated protocols could be found, no significant differences between commercial and non-commercial sponsors were detected. Furthermore, the average duration for the approval of all substantial amendments of an integrated trial protocol varies significantly depending on the primary and secondary endpoints of the trial

*SAD* single ascending dose trials, *MAD* multiple ascending dose trials, *SD/SS* trials using stable dose(s) (single or multiple) or steady-state conditions, *PD* trials with primary pharmacodynamic objectives

*p < 0.001 (integrated protocols compared to non-integrated)

^a^Percentage refers to integrated protocols only

It became obvious that oncology trials were the most complex of all integrated trials with the longest time to initial authorisation, the largest number of substantial amendments and the longest respective approval time for all amendments of the clinical trial application (Tables 1, 2 and 3).

### Questionnaire

From the total of 327 applicants invited, 95 participated in the online survey (29.1%). Forty-nine participants (51.6%) completed the entire survey questionnaire, and 46 participants (48.4%) terminated the online survey before responding to all of the questions asked. Only the most prominent and most interesting answers to the survey questions are presented here; the complete questionnaire results are provided in the electronic supplement.

**Fig. 5:**
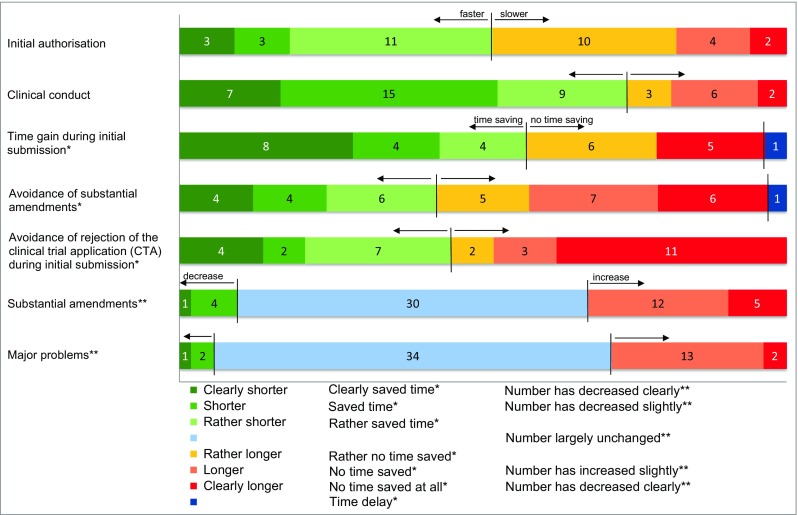
Survey results on duration of initial authorisation and clinical conduct, time-saving factors, and number of substantial amendments and major problems. Survey participants were asked to rate to what extent the duration of initial authorisation and clinical conduct of a CTA have changed when using integrated protocols compared to standard protocols. An ascending six-point scale ranging from ‘clearly shorter’ to ‘clearly longer’ was used. The first three options (‘clearly shorter’, ‘shorter’and ‘rather shorter’) were classified as faster initial authorisation and faster clinical conduct, whereas the last three options (‘rather longer’, ‘longer’ and ‘clearly longer’) were classified as slower initial authorisation and slower clinical conduct (bar nos. 1 and 2). Moreover, the survey participants were asked to rate the impact of the three aspects ‘Time gain during initial submission’, ‘Avoidance of substantial amendments’ and ‘Avoidance of rejection of the CTA during initial submission’ on time-saving when using integrated protocols. An ascending six-point scale ranging from ‘clearly saved time’ to ‘no time saved at all’ was used. The first three options (‘clearly saved time’, ‘saved time’ and ‘rather saved time’) were classified as time-saving factors, whereas the last three options (‘rather no time saved’, ‘no time saved’ and ‘no time saved at all’) were classified as no time-saving factors. Additionally, the participants could also choose the option ‘time delay’, when time was lost due to use of integrated protocols (bar nos. 3, 4 and 5). Finally, the survey participants were asked to rate to what extent the numbers of substantial amendments and major problems have changed when using integrated protocols compared to standard protocols. An ascending five-point scale ranging from ‘number has decreased clearly’ to ‘number has increased clearly’ was used. The first two options (‘number has decreased clearly’ and ‘number has decreased slightly’) were classified as decrease in substantial amendments and major problems, whereas the last two options (‘number has increased slightly’ and ‘number has increased clearly’) were classified as increase. Additionally, the participants could also choose the option ‘number largely unchanged’ when no relevant differences in the number of substantial amendments and major problems were observed (bar nos. 6 and 7)

The majority of participants (70.5%) reported their sponsor status as ‘commercial sponsor’. Most of them describe their institution as pharmaceutical industry, some as contract research organisations. Most of the ‘non-commercial sponsors’ are considered academic institutions (e.g. university hospitals). 63.2% of the respondents have been working in the field of clinical trials since before the year 2004. The majority of applicants use integrated protocols in up to 30% of their early-phase clinical trials; only less than 20% reported using integrated protocols even more frequently. Nearly one third of the applicants are planning to use integrated phase I and I/II protocols more frequently in the future, whereas another one third are not planning on doing so or was undecided when participating in the survey. Interestingly, only 36.8% of the applicants are aware of the possibility of accelerated authorisation procedures (14 days) for follow-up phase I clinical trial applications according to Sections 8 and 9 of the German Ordinance on Good Clinical Practice. Consequently, only 21.1% have ever made use of this accelerated authorisation process. Overall, in the applicants’ opinion, the preparation of integrated phase I and I/II clinical trials is not more extensive than the preparation of non-integrated clinical trials. 30.5% say the preparation is (‘at least rather’) not more extensive, while 20.0% say it is (‘at least rather’) more extensive. 32.6% of the applicants believe that the overall time for conducting a clinical trial following an integrated protocol is shorter compared to an early-phase clinical trial development programme with non-integrated protocols, while only 11.6% considered integrated protocol approaches to be prolonged. 44.2% of the participants considered the number of substantial amendments to be largely unchanged or only slightly increased when choosing integrated early-phase clinical trials in comparison with non-integrated trials (Fig. 5). Besides, 49.5% stated that this trend is equal for the number of major problems (major incidents of different types during trial conduct). With respect to the absolute number of submitted substantial amendments per protocol, the participants reported a range from 0 to > 5 with a median of 2.

**Fig. 6:**
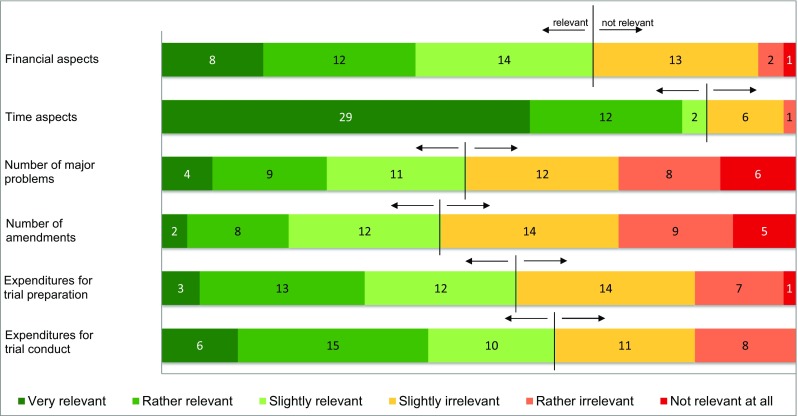
Survey results on motivation. Relevance ratings of motivational aspects for using integrated protocols. Survey participants were asked to rate to what extent ‘Financial aspects’, ‘Time aspects’, ‘Number of major problems’, ‘Number of substantial amendments’, ‘Expenditures for trial preparation’ and ‘Expenditure for trial conduct’ are relevant as motivation for using integrated protocols. An ascending six-point scale ranging from ‘very relevant’ to ‘not relevant at all’ was used. The first three options (‘very relevant’, ‘rather relevant’ and ‘slightly relevant’) were classified as relevant for motivation for using integrated protocols, whereas the last three options (‘slightly irrelevant’, ‘rather irrelevant’ and ‘not relevant at all’) were classified as not relevant for using integrated protocols

The most prevalent motivation for using integrated protocols was the aspect of saving money. Interestingly, only 36.8% of the participants believe that the use of integrated protocols really reduces costs while 7.4% think that using integrated protocols even increases costs. The vast majority (93.3%) of those respondents who considered financial aspects as relevant motivation for using integrated protocols believe that the use of such protocols is clearly money-saving (*p* = 0.006). Particularly, commercial sponsors focused on the time-saving aspect, too (Fig. 6). 38.9% of the respondents considered the use of integrated protocols as more time-saving compared to standard approach, while only 8.4% consider them to be more time-consuming in the overall trial conduct. In contrast to non-commercial sponsors, 42 out of 46 survey participants who identified themselves as commercial sponsors judged time aspects as a highly relevant factor for using integrated protocols (*p* = 0.003). In all, there was a very significant interrelation between money-saving and time-saving aspects when asked for the motivation of using integrated protocols (*p* < 0.001). The majority of applicants preferring integrated protocols believe that the time gain is mainly based on a shorter duration until initial authorisation (Fig. 5). Sixteen out of 27 participants who claim that integrated protocols save time are of the opinion that the duration until initial authorisation after submission of an integrated CTA to the BfArM is shorter compared to that for non-integrated protocols (*p* = 0.015).

## Discussion

In our study, we analysed 2969 phase I and I/II CTAs according to their protocol type and sponsor status. Furthermore, we investigated the duration of the authorisation process as well as the subsequent regulatory activities of integrated protocols in comparison with standard protocols. As almost all phase I and I/II clinical trials submitted to the BfArM were included in the analysis, it was the first time that such information was analysed to this great an extent. Overall, only sparse information is available on this topic so far [21]. We could prove that the assumed trend towards a more frequent use of integrated protocols [13] clearly exists with an increase of approximately 10% (from 17.9% to more than 28% integrated protocols) in 10 years (in Germany). Although the vast majority (~ 90%) of clinical trials are conducted by commercial sponsors such as pharmaceutical industry or contract research organisations, both commercial and non-commercial sponsors use integrated protocols in almost the same ratio.

We could confirm the internal hypothesis that integrated protocols are more complex [14, 22] and therefore more difficult in the authorisation process [21]. It could clearly be shown that integrated protocols require significantly longer initial authorisation times (58 vs. 53 days) which can partly be attributed to a significantly larger number of deficiency issues (56 vs. 46%). Furthermore, the internal hypothesis that integrated protocols lead to increased subsequent regulatory activities [6, 21, 23] could be confirmed. We could clearly show that the use of integrated protocols leads to a significantly larger number of substantial amendments (1.9 vs. 1.2 per clinical trial (CT)). Additionally, the total duration for the approval process of all amendments of a CTA is significantly longer in integrated protocols (22 days) compared to standard protocols (14 days).

Most of these findings are in line with the results from the online questionnaire. Since the vast majority of the participants were commercial sponsors with many years of experience in the field of clinical trials, we can say that in this analysis, mainly experts commented on the issue. It became clear that not only the participants use integrated protocols in up to 30% of their clinical trials nowadays, but also that one third of them are intending to use integrated protocols even more frequently in the future. It was surprising that only by 36% of the respondents and not unanimously financial and temporal aspects were rated to be the key motivations for using integrated protocols in entire phase I development programmes. Anyhow, there are also results from the online survey which contradict our findings from the database analysis. For example, the majority of survey participants claimed that better time efficiency in integrated protocols is achieved through a shorter period until initial authorisation of a CTA. Moreover, it was stated that the number of substantial amendments in clinical trials following integrated protocols was equal to the number in standard protocols. As discussed above, these findings could not be confirmed by our database analysis. It could be possible that the experience with integrated protocols or the sponsor status has an influence on the point of view of the participants. This way, it would be comprehensible if very experienced applicants are of the opinion that they do not encounter problems like time loss during initial authorisation or increased numbers of substantial amendments. Finally, to emphasise one particular aspect, the survey indicated that the intended acceleration with a 14-day authorisation process for follow-up CTs as provided by Sections 8 (3) and 9 (3) of the German Ordinance on Good Clinical Practice [24–26] is hardly established and is virtually not used. A possible explanation could be that this is a national ordinance which is unknown in other member states of the EU. However, this option can be considered as an alternative for using integrated protocols [4, 13].

The efforts for preparation and filing of complex CT protocols just like their processing time during assessment by the competent federal higher authority actually last longer [5, 14, 27] as compared to standard protocols. However, it is important to consider that integrated protocols combine at least two, three or even more standard phase I (I/II) protocols [4, 6–9, 28]. Thus, clinical trials which would have been conducted separately, consecutively and independently can be conducted in parts simultaneously, straight successively or overlapping [5, 7, 9, 29]. Due to the reduced total number of clinical trials needed to collect the same data and therefore the reduced interaction time with authorities and ethics committees, the total time from first application to final results *may be* substantially decreased [5, 30].

Unfortunately, for both regulatory authorities and ethics committees, it is difficult that in CTs with integrated protocols, they are frequently requested to decide on all parts of a CTA without knowledge of the results of the single-trial parts. In fact, integrated phase I protocols are more of a series of mandatory stand-alone clinical trials than logical parts of a single clinical trial. For example for the assessment of an integrated protocol, authorities and ethics committees are requested to authorise the multiple ascending dose (MAD) part before receiving results from the single ascending dose (SAD) part. At this time, neither the competent authority nor the ethics committee has any safety information stemming from the use in humans regarding the new medicinal product. One possibility to solve this dilemma is the voluntary interruption of an integrated clinical trial after each study part. The study will not be continued until all relevant data from the preceding trial part (e.g. the SAD part) including safety information have been evaluated. In order to continue with the study, the applicant has to submit these data per substantial amendment to the competent authority as well as to the ethics committee for authorisation. However, many applicants avoid this approach due to time issues. Commonly, they favour a notification to the competent authorities and ethics committees (‘tell-and-do’ approach). This, however, imposes an undue time pressure on both the competent authority and ethics committee. On the other hand, this implies a role change where competent authorities and ethics committees become ‘real-time’ guards responsible for tasks that would normally lie with the sponsor. Therefore, it could be noted that more work, in particular responsibility and follow-up, is pushed over from the sponsors to the authorities and ethics committees.

At this point, both competent authorities and ethics committees find themselves in a conflict of interest. On the one hand, the authorisation of clinical trials shall not be delayed by unnecessary bureaucratic burden; on the other hand, the safety, protection and well-being of the trial subjects must be ensured by authorisation and assessment processes under the (German) Medicinal Products Act and the (German) Ordinance on Good Clinical Practice [4, 13, 31]. One option to escape from this dilemma could be clearly defined decision-making processes and ‘no-go’ criteria in clinical trial protocols. As long as clear decision rules are prespecified and predefined limits in terms of safety and exposure are not exceeded during conduct, progression to the subsequent trial part without interruption and reporting *could be* acceptable. In contrast, the clinical trial *must be* interrupted and a substantial amendment with detailed assessment of the events *must be* submitted when leaving this ‘safety corridor’.

At the present time, the higher efforts in reviewing and assessing integrated or otherwise complex trial protocols are neither covered by the current legislation [12] nor by the upcoming EU regulation no. 536/2014 [32]. The BfArM reviews CTAs in three parallel processes, assessing the investigational product dossier(s), the investigator’s brochure(s) and the trial protocol. While the dossiers and the investigator’s brochures are more or less comparable between standard protocols and integrated protocols, the assessment of the clinical trial protocol is substantially more complex and more time-consuming for integrated phase I and I/II clinical trial protocols. Therefore, BfArM increased the fees for the assessment of integrated protocols by approx. 25% per subtrial in addition to the basic fee since March 2015 [15] to cover the additional expenses, as BfArM is mainly fee-financed. Nevertheless, it could be assumed that this compensation is not implemented by all ethics committees which often do not distinguish between standard and integrated protocols. If so, it could certainly be argued that the society pays for the increased work and costs, at least partially.

So far, new and adaptive study designs have been used primarily in phase II and III clinical trials [27, 28, 30, 33]. Now, they are better and better finding their way into early-phase (I/II) clinical trials. We could clearly show that the use of integrated protocols does in fact also imply some disadvantages. However, numerous experts in this field as well as the majority of our online survey participants agree that these disadvantages are outweighed by the advantages of the entire phase I development programme. Regulatory bodies are already reacting to the increasing use of integrated protocols in early-phase clinical trials. For instance, the 2017 update of the EMA’s phase I guideline has introduced a new section providing basic guidance on requirements, legal regulations and sequence of CTs following integrated protocols [34].

### Limitations

In our analysis, only single clinical trial protocols and not complete phase I development programmes could be investigated, because the latter are not necessarily conducted in one country only. Therefore, we could only speculate about potential analogies regarding development programmes with the information deduced from the single CTAs. Another limitation is that the analysis only included the CTAs submitted to the BfArM. Nevertheless, this covers approximately 90% of all phase I trial protocols during the period of our analysis in Germany. As all applicants who submitted at least one integrated protocol during the observation period were invited to participate in the survey, the return rate of 29% nevertheless represents a substantial proportion of the entire sponsor population using integrated protocols. It might be that due to the length of the survey and the relatively large number of mandatory questions, not every questionnaire was completed. Only 52% of the participants answered every question.

## Conclusion and outlook

We could confirm an obvious and steady increase in the number of integrated protocols submitted to the BfArM in the past years for phase I CTs. The major motivation for this strategy is the chance to increase efficiency in time and costs. An ‘all-in-one’ phase I CT following an integrated protocol *may be* completed faster and more (cost) efficiently, although *parts* of the conduct *are* more time-consuming and more expensive compared to non-integrated protocols. The advantages for applicants, however, prove to be critical for competent authorities and ethics committees. As integrated protocols will be implemented increasingly in early-phase clinical trials, competent authorities as well as ethics committees cannot buck this trend categorically. On the one hand, they are forced to adapt to the changing development concepts while they must maintain their responsibilities and duties on the other hand. A feasible solution would be to implement harmonised prespecified decision-making algorithms in integrated protocols, e.g. in which cases it is necessary to submit a substantial amendment as opposed to a notification. The more specified and clearer the decision-making process is defined in the trial protocol, the easier it is for competent authorities and ethics committees to give approval without insisting on interim results and substantial amendments. With the help of protocol templates adjusted to various research demands, this approach could be facilitated and approvals could be accelerated. This would benefit all, applicants, competent authorities, ethics committees and trial subjects.

## Electronic supplementary material

ESM 1 (PDF 788 kb)
