# Supplementary material for: Analysis of integrated clinical trial protocols in early phases of medicinal product development
Source: Eur J Clin Pharmacol. 2017 Sep 18;73(12):1565–77. doi: 10.1007/s00228-017-2335-y (PMC5684304; doi:10.1007/s00228-017-2335-y)
Supplement: Supplementary file 1 — (PDF 788 kb) [file 228_2017_2335_MOEMS1_ESM.pdf]

Analysis of Integrated Clinical Trial Protocols in Early Phases of Medicinal Product Development

European Journal of Clinical Pharmacology

Kevin Fruhner, Gunther Hartmann, Thomas Sudhop

K. Fruhner

Institute of Clinical Chemistry and Clinical Pharmacology, University Hospital, University of Bonn, Sigmund-Freud-Straße 25, 53127 Bonn, Germany

e-mail: kevin.fruhner@uni-bonn.de

G. Hartmann

Institute of Clinical Chemistry and Clinical Pharmacology, University Hospital, University of Bonn, Sigmund-Freud-Straße 25, 53127 Bonn, Germany

e-mail: gunther.hartmann@ukb.uni-bonn.de

T. Sudhop

Federal Institute for Drugs and Medical Devices (BfArM), Kurt-Georg-Kiesinger-Allee 3, 53175 Bonn, Germany

e-mail: thomas.sudhop@bfarm.de

Electronic Supplement / Entire online survey questionnaire All questions from the questionnaire are presented in consecutive order. Every answer is included with results in total numbers as well as in percentage. Additionally the responses to every question are shown in a bar diagram.

Results from the online survey questionnaire

Number of records in this query:	95
Total records in survey:	95
Percentage of total:	100.00%

Field summary for Q1

Is your institution considered to be a commercial sponsor?

Answer	Count	Percentage
Yes (Y)	67	70.53%
No (N)	7	7.37%
No answer	13	13.68%
Not completed or Not displayed	8	8.42%

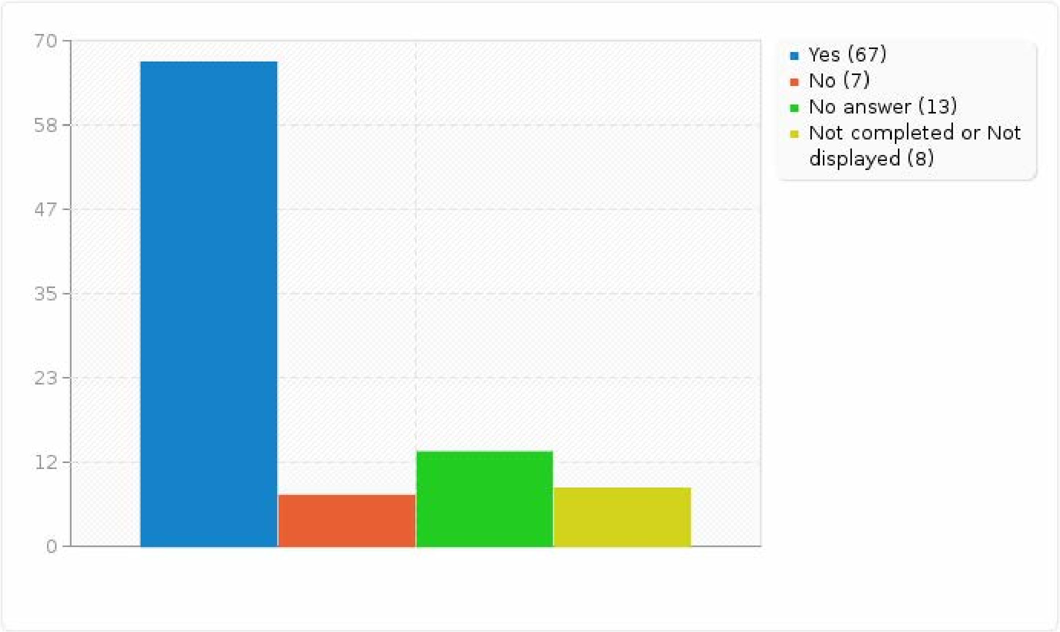

Field summary for Q2

For how long has your institution been conducting phase 1 clinical trials?

Answer	Count	Percentage
before 2004 (A1)	60	63.16%
since 2004 (A2)	1	1.05%
since 2005 (A3)	0	0.00%
since 2006 (A4)	2	2.11%
since 2007 (A5)	0	0.00%
since 2008 (A6)	2	2.11%
since 2009 (A7)	4	4.21%
since 2010 (A8)	3	3.16%
since 2011 (A9)	0	0.00%
since 2012 (A10)	0	0.00%
since 2013 (A11)	0	0.00%
since 2014 (A12)	0	0.00%
No answer	1	1.05%
Not completed or Not displayed	22	23.16%

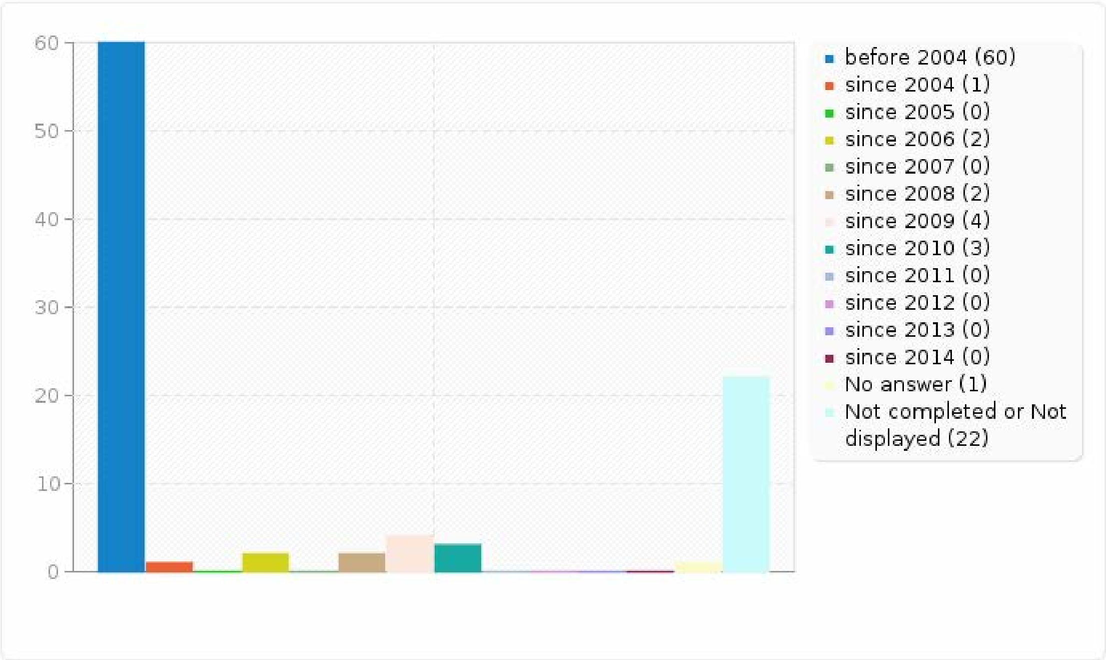

Field summary for Q3

What is the approximate percentage of integrated phase 1 protocols as compared to the total number of phase 1 clinical trials your institution conducted in the past 5 years?

Answer	Count	Percentage
< 10% (A1)	18	18.95%
10%-30% (A2)	17	17.89%
30%-50% (A3)	10	10.53%
>50% (A4)	7	7.37%
No answer	14	14.74%
Not completed or Not displayed	29	30.53%

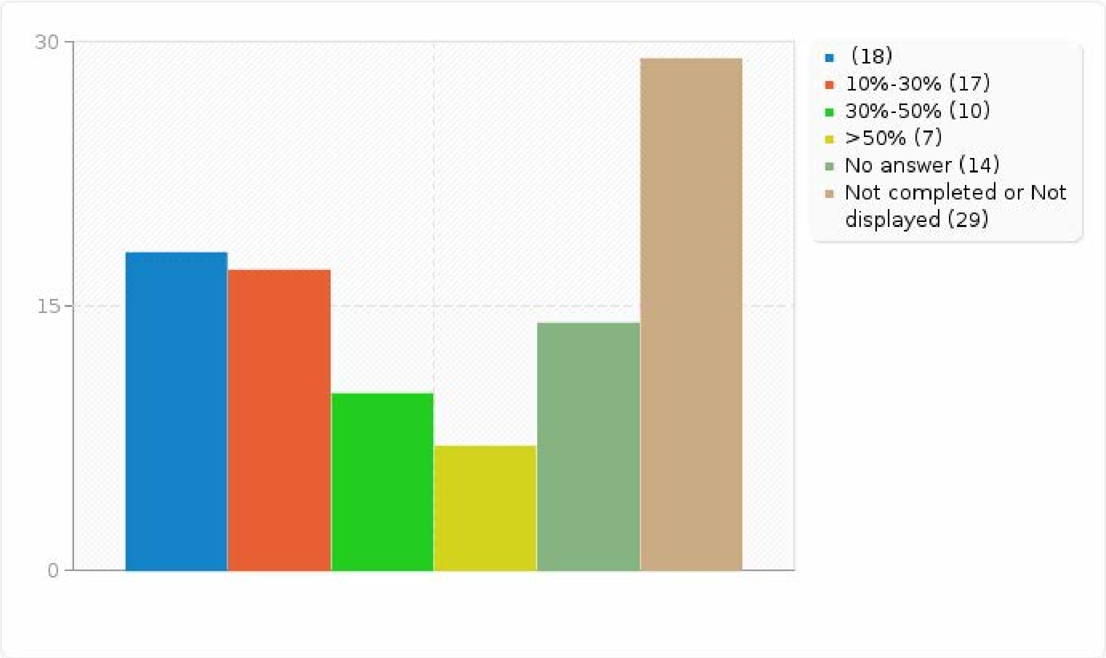

Field summary for Q4

Is your institution planning to use integrated phase 1 protocols more frequently in the future?

Answer	Count	Percentage
Yes (A1)	31	32.63%
No (A2)	7	7.37%
Do not know (A3)	24	25.26%
No answer	2	2.11%
Not completed or Not displayed	31	32.63%

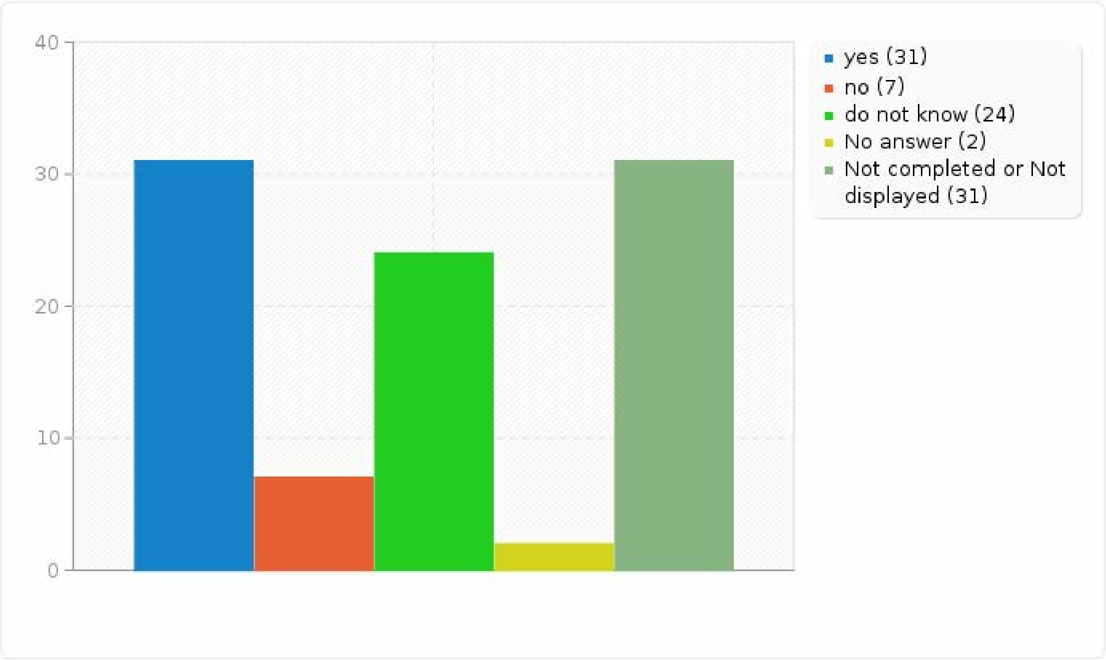

Field summary for Q5

Are you aware of the possibility of accelerated authorisation procedures (14 days) for follow-up phase 1 clinical trial applications [cf.: Section 8 (3) and 9 (3) of the German Ordinance on Good Clinical Practice, GCP-Verordnung]

Answer	Count	Percentage
Yes (Y)	35	36.84%
No (N)	28	29.47%
No answer	0	0.00%
Not completed or Not displayed	32	33.68%

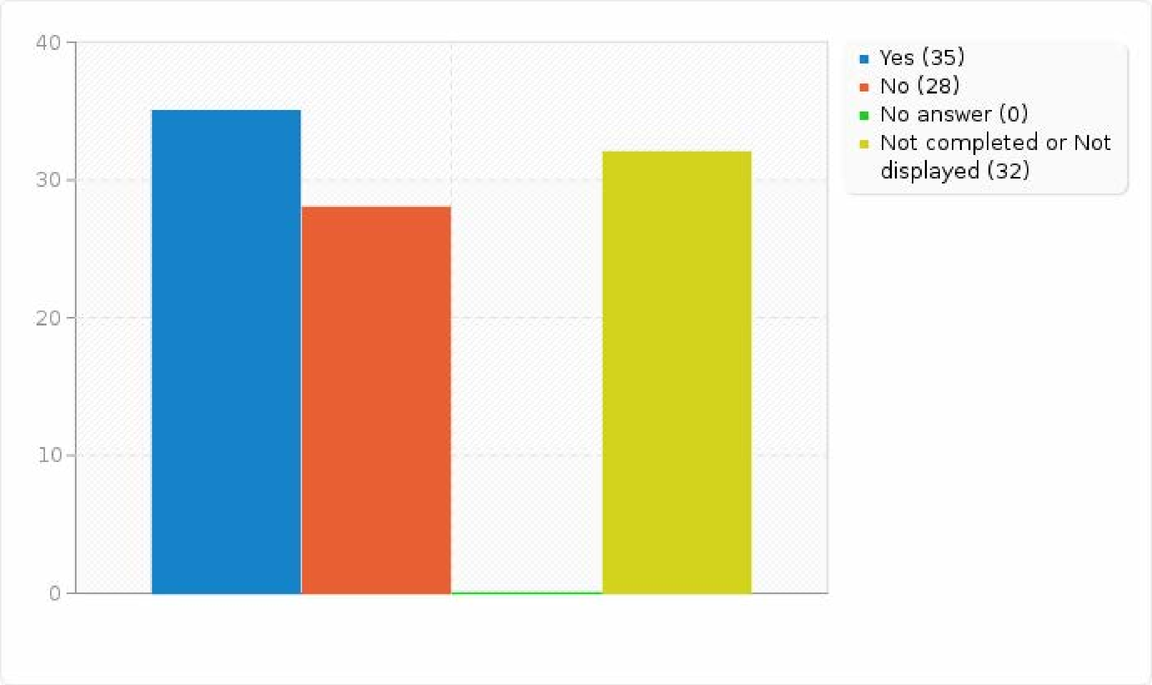

Field summary for Q6

Has your institution already made use of the aforementioned accelerated authorisation process (14 days) in accordance with Section 8/9 (3) of the German Ordinance on Good Clinical Practice (GCP-Verordnung)?

Answer	Count	Percentage
Yes (Y)	20	21.05%
No (N)	41	43.16%
No answer	0	0.00%
Not completed or Not displayed	34	35.79%

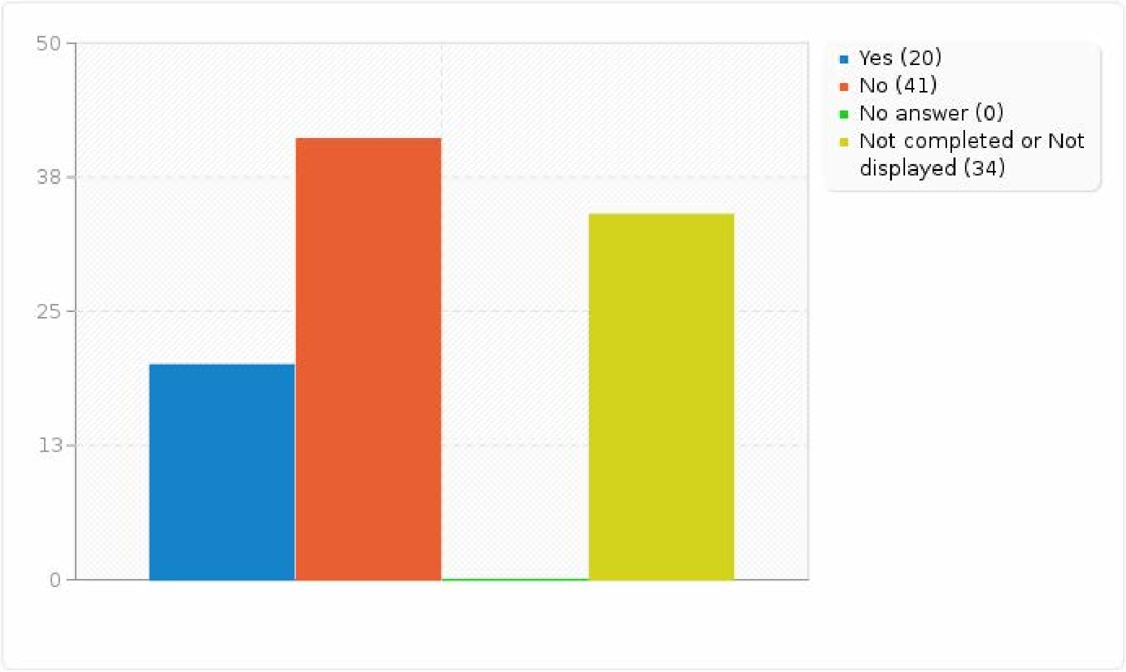

Field summary for Q7

Please rate how successfully your institution has already made use of the aforementioned accelerated authorisation process in accordance with Section 8/9 (3) of the German Ordinance on Good Clinical Practice (GCP-Verordnung). If you have answered the preceding questions with "no", please select "no answer"!

Answer	Count	Percentage
Not successfully at all (A1)	0	0.00%
Not successfully (A2)	2	2.11%
Rather not successfully (A3)	3	3.16%
Rather successfully (A4)	1	1.05%
Successfully (A5)	8	8.42%
Very successfullly (A6)	3	3.16%
No answer	42	44.21%
Not completed or Not displayed	36	37.89%

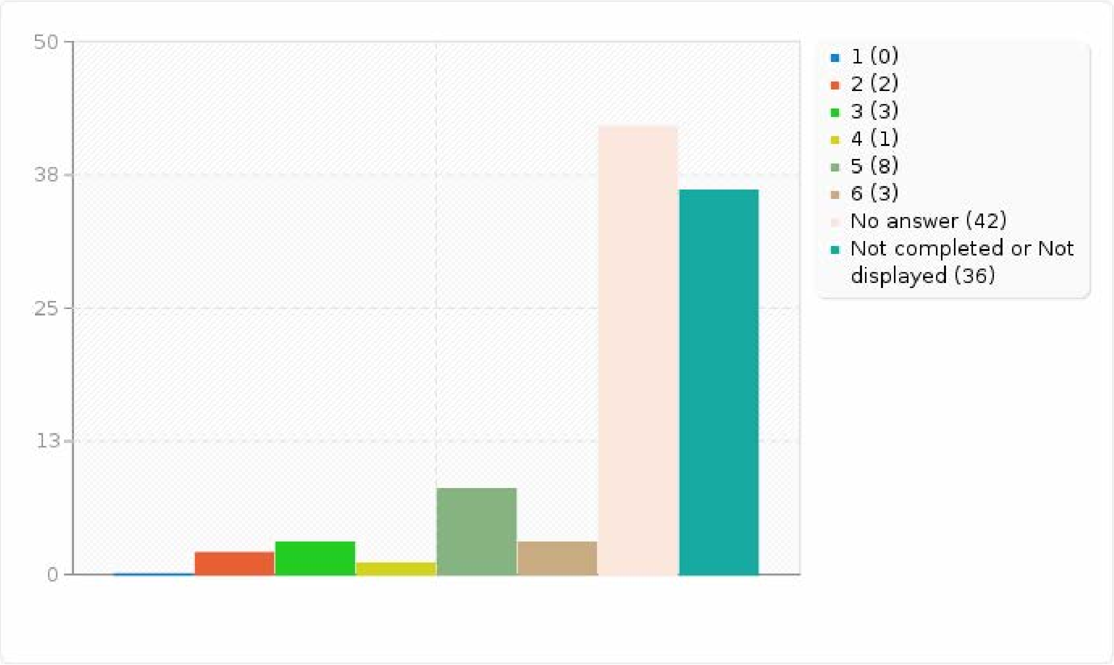

Field summary for Q8

In integrated protocols, several objectives are investigated in parallel/simultaneously where several classical phase 1 clinical trials would be necessary to clarify the same objectives. In your experience, is the preparation of one integrated protocol generally more extensive than the otherwise necessary preparation of several protocols for classical phase 1 clinical trials?

Answer	Count	Percentage
Not more extensive at all (A1)	9	9.47%
Rather less extensive (A2)	11	11.58%
Slightly less extensive (A3)	9	9.47%
Slightly more extensive (A4)	5	5.26%
Rather more extensive (A5)	9	9.47%
Clearly more extensive (A6)	5	5.26%
No answer	10	10.53%
Not completed or Not displayed	37	38.95%

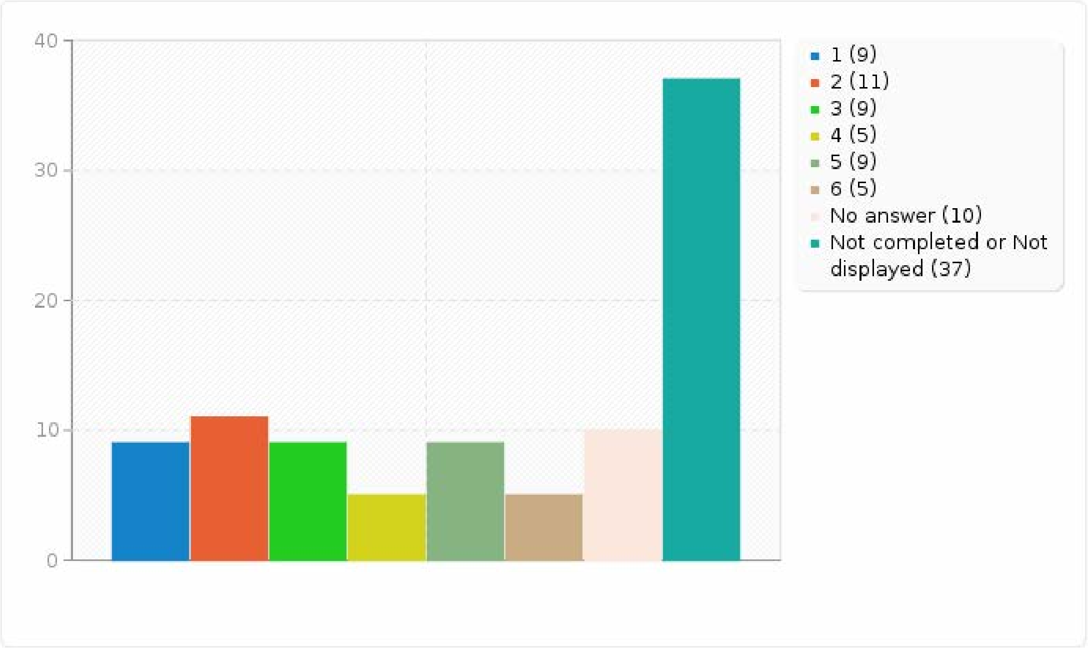

Field summary for Q9

How does the recruitment of subjects for an integrated protocol compare to that in corresponding classical phase 1 clinical trials?

Answer	Count	Percentage
Clearly more difficult (A1)	3	3.16%
Rather more difficult (A2)	3	3.16%
Slightly more difficult (A3)	12	12.63%
Slightly less difficult (A4)	19	20.00%
Rather less difficult (A5)	1	1.05%
Clearly less difficult (A6)	1	1.05%
No answer	18	18.95%
Not completed or Not displayed	38	40.00%

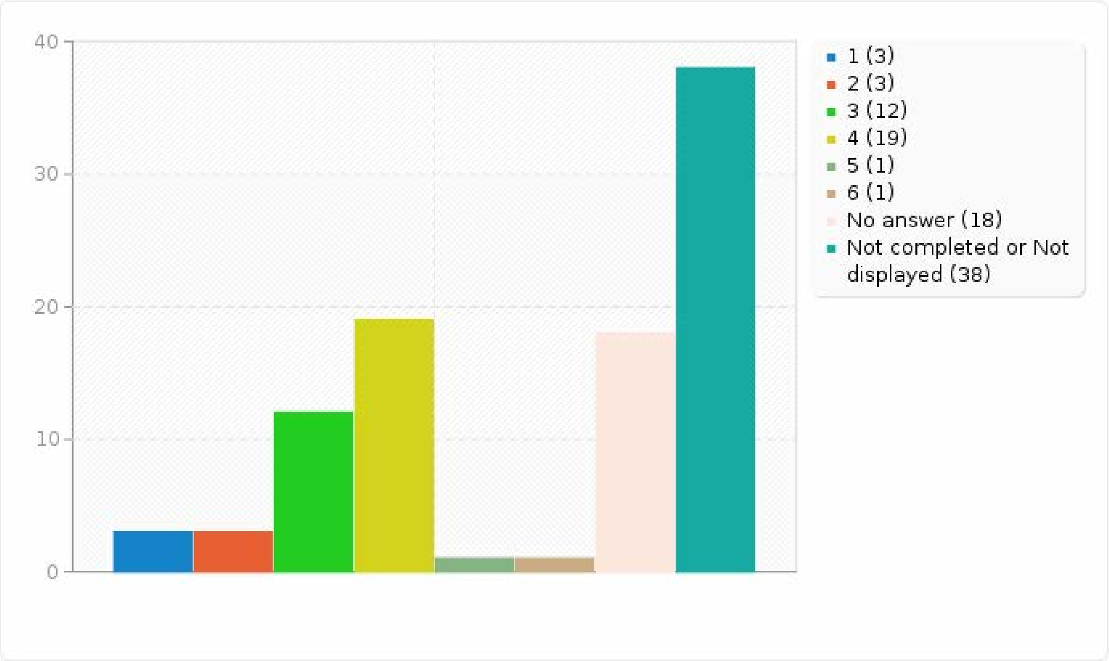

Field summary for Q10

Please rate the duration of the clinical conduct of a clinical trial with an integrated protocol as compared to the duration of clinical trial sets with non-integrated protocols.

Answer	Count	Percentage
Clearly shorter (A1)	7	7.37%
Rather shorter (A2)	15	15.79%
Slightly shorter (A3)	9	9.47%
Slightly longer (A4)	3	3.16%
Rather longer (A5)	6	6.32%
Clearly longer (A6)	2	2.11%
No answer	13	13.68%
Not completed or Not displayed	40	42.11%

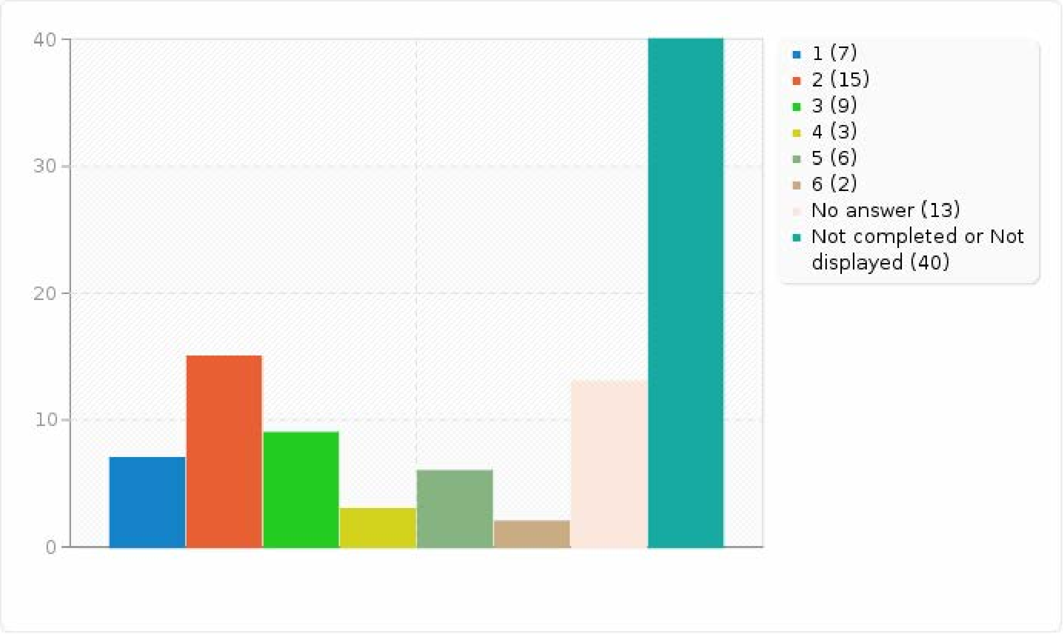

Field summary for Q11

In integrated protocols, several objectives are investigated in parallel/simultaneously where otherwise several classical phase 1 clinical trials would be necessary to clarify the same objectives. In your experience, what is the duration until initial authorisation of an integrated protocol by the BfArM as compared to the similar classical phase 1 clinical trials?

Answer	Count	Percentage
Clearly shorter period of time (A1)	3	3.16%
Rather shorter period of time (A2)	3	3.16%
Slightly shorter period of time (A3)	11	11.58%
Slightly longer period of time (A4)	10	10.53%
Rather longer period of time (A5)	4	4.21%
Clearly longer period of time (A6)	2	2.11%
No answer	22	23.16%
Not completed or Not displayed	40	42.11%

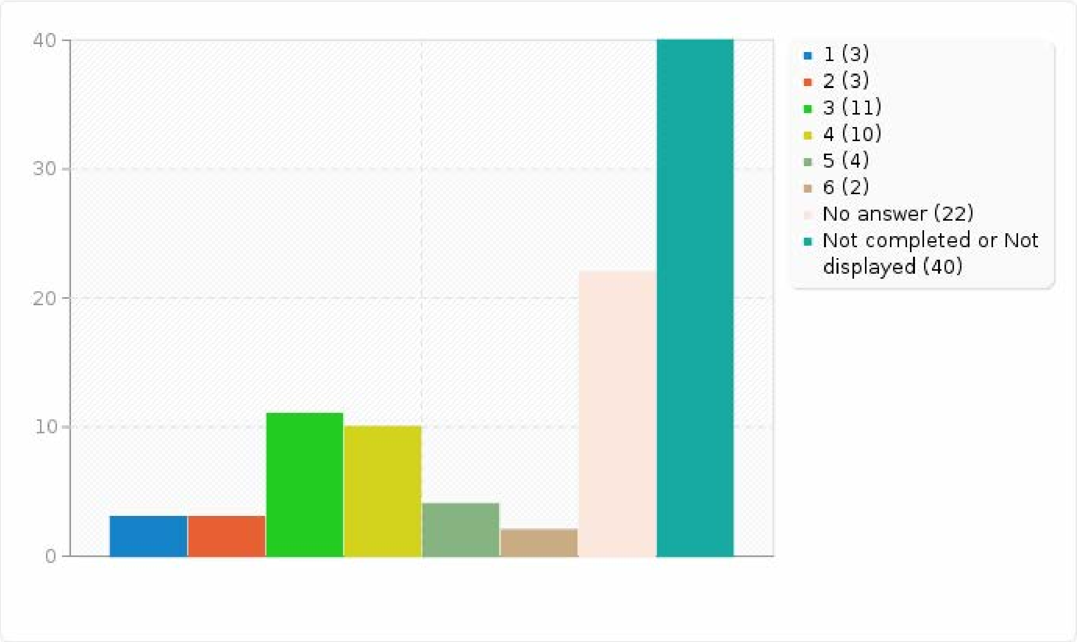

Field summary for Q12

In your experience, how has the number of substantial amendments changed in connection with integrated phase 1 protocols as compared to classical phase 1 clinical trials?

Answer	Count	Percentage	Sum
Number has decreased clearly (A1)	1	1.08%	5.38%
Number has decreased slightly (A2)	4	4.30%	
Number largely unchanged (A3)	30	32.26%	32.26%
Number has increased slightly (A4)	12	12.90%	
Number has increased clearly (A5)	5	5.38%	18.28%
Sum (Answers)	52	100.00%	100.00%
Number of cases	54	100.00%	
No answer	2	2.11%	
Not completed or Not displayed	41	43.16%	

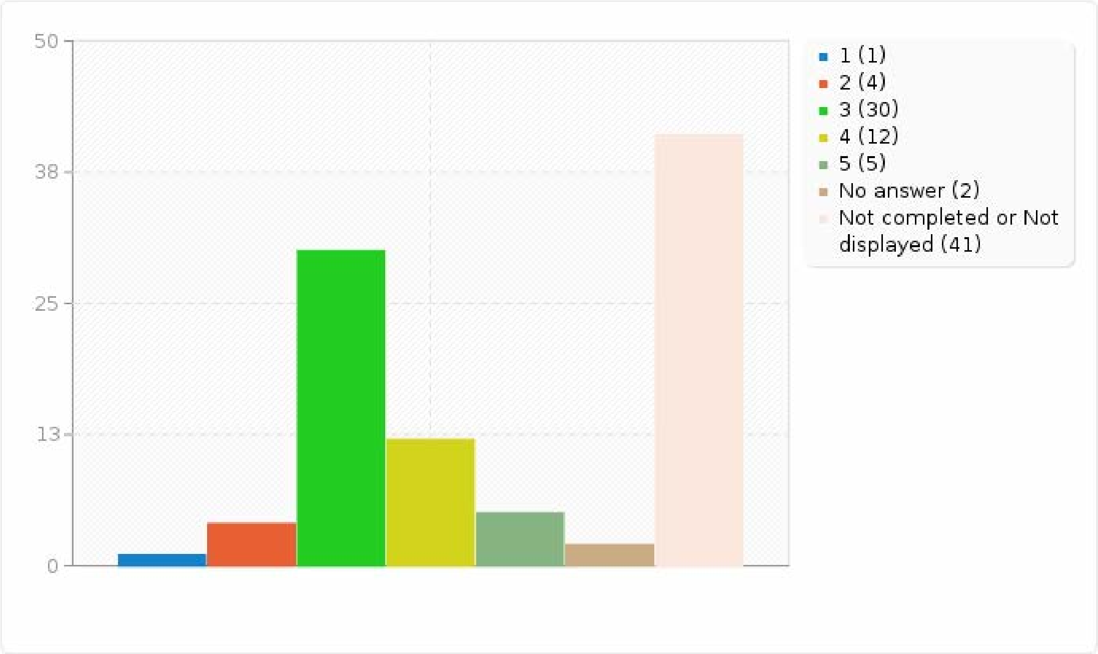

Field summary for Q13

In your experience, how has the number of major problems varied when conducting trials with integrated protocols as compared to classical phase 1 clinical trials?

Answer	Count	Percentage	Sum
Number has decreased clearly (A1)	1	1.05%	3.16%
Number has decreased slightly (A2)	2	2.11%	
Number largely unchanged (A3)	34	35.79%	35.79%
Number has increased slightly (A4)	13	13.68%	
Number has increased clearly (A5)	2	2.11%	15.79%
Sum (Answers)	52	100.00%	100.00%
Number of cases	52	100.00%	
No answer	0	0.00%	
Not completed or Not displayed	43	45.26%	

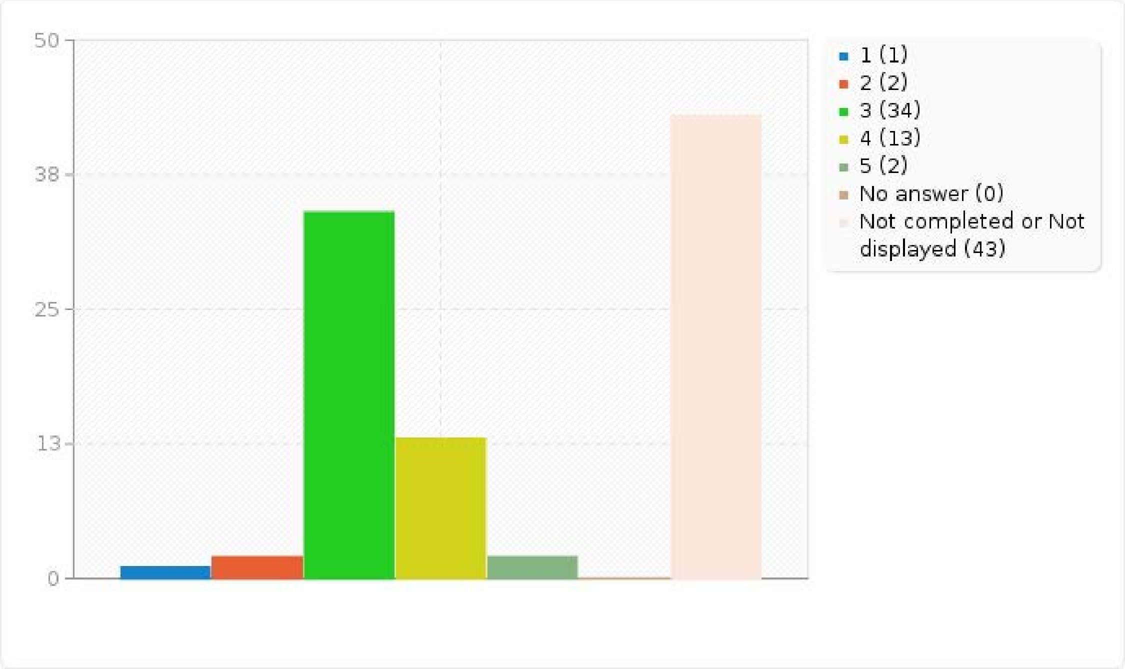

Field summary for Q14

All in all, has using integrated protocols resulted in your institution saving time as compared to conducting equivalent classical phase 1 clinical trials?

Answer	Count	Percentage
Clearly saved time (A1)	14	14.74%
Rather saved time (A2)	11	11.58%
Slightly saved time (A3)	12	12.63%
Slight loss of time (A4)	6	6.32%
Rather loss of time (A5)	1	1.05%
Clear loss of time (A6)	1	1.05%
No answer	6	6.32%
Not completed or Not displayed	44	46.32%

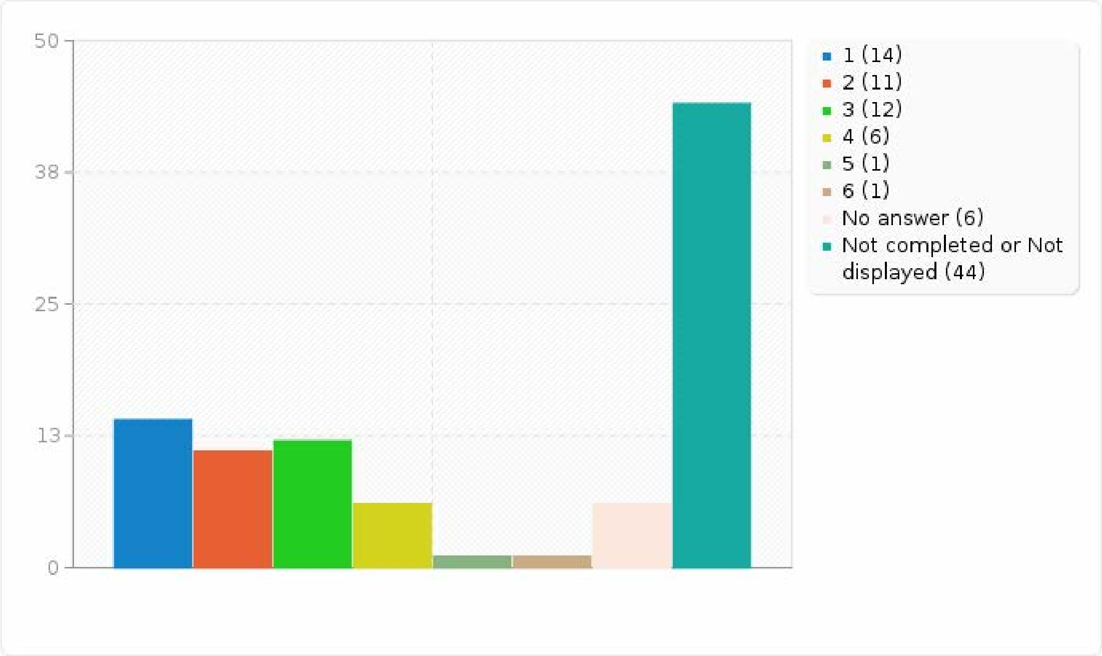

Field summary for Q15

All in all, has using integrated protocols resulted in your institution saving money as compared to conducting equivalent classical phase 1 clinical trials?

Answer	Count	Percentage
Clearly saved money (A1)	9	9.47%
Rather saved money (A2)	11	11.58%
Slightly saved money (A3)	15	15.79%
Slightly increased expenses (A4)	6	6.32%
Rather increased expenses (A5)	1	1.05%
Clearly increased expenses (A6)	0	0.00%
No answer	9	9.47%
Not completed or Not displayed	44	46.32%

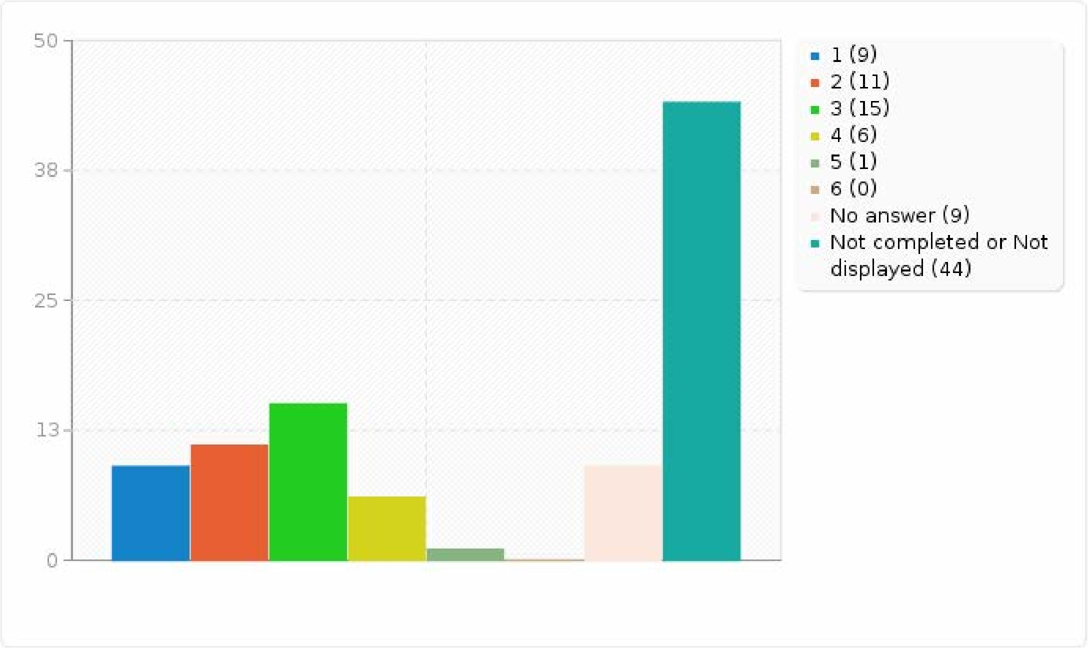

Field summary for Q16.1

Please rate to what extent the following aspects are important for the use of integrated protocols from the sponsor´s point of view.
[Financial aspects]

Answer	Count	Percentage
Not relevant at all (A1)	1	1.05%
Rather irrelevant (A2)	2	2.11%
Slightly irrelevant (A3)	13	13.68%
Slightly relevant (A4)	14	14.74%
Rather relevant (A5)	12	12.63%
Very relevant (A6)	8	8.42%
No answer	1	1.05%
Not completed or Not displayed	44	46.32%

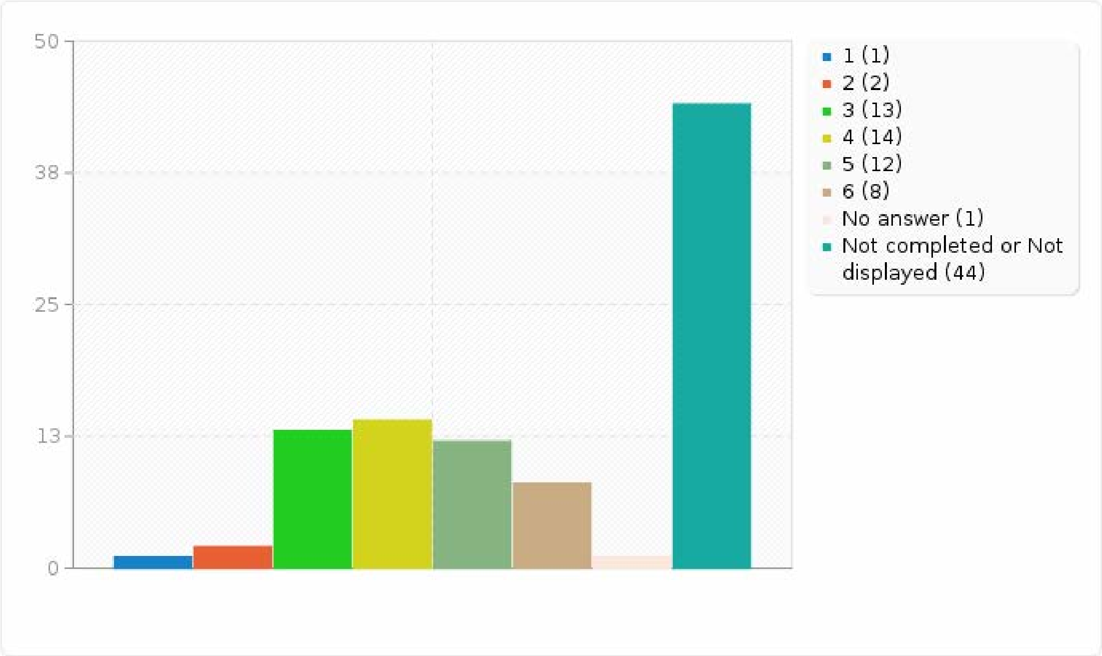

Field summary for Q16.2

Please rate to what extent the following aspects are important for the use of integrated protocols from the sponsor´s point of view.
[Time aspects]

Answer	Count	Percentage
Not relevant at all (A1)	0	0.00%
Rather irrelevant (A2)	1	1.05%
Slightly irrelevant (A3)	6	6.32%
Slightly relevant (A4)	2	2.11%
Rather relevant (A5)	12	12.63%
Very relevant (A6)	29	30.53%
No answer	1	1.05%
Not completed or Not displayed	44	46.32%

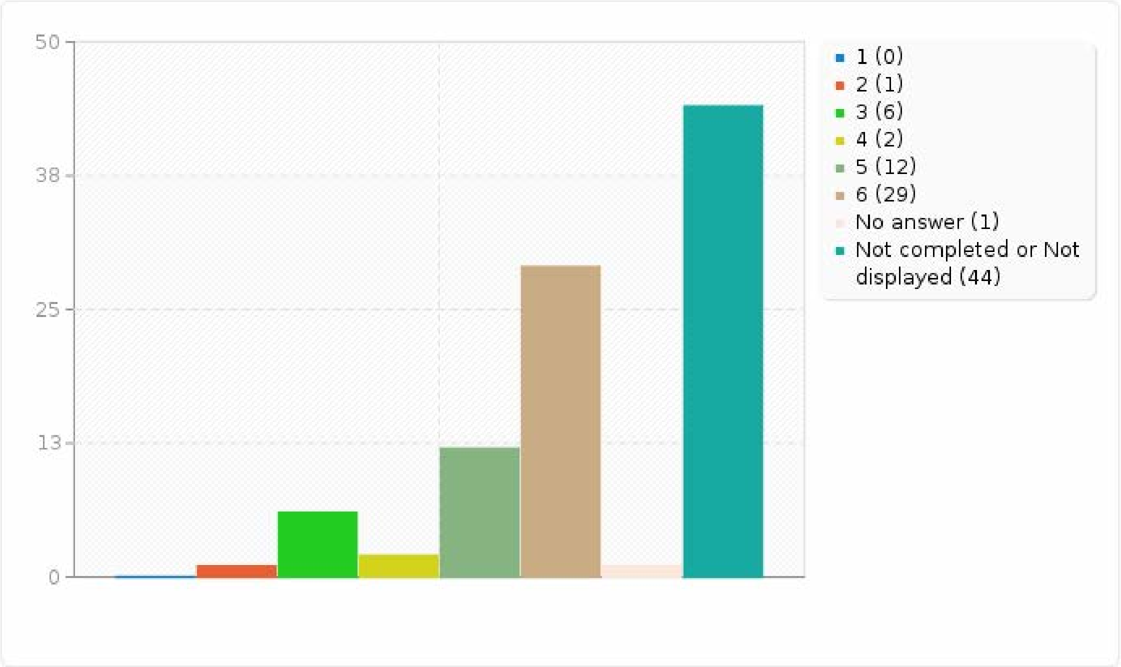

Field summary for Q16.3

Please rate to what extent the following aspects are important for the use of integrated protocols from the sponsor´s point of view.
[Number of major problems]

Answer	Count	Percentage
Not relevant at all (A1)	6	6.32%
Rather irrelevant (A2)	8	8.42%
Slightly irrelevant (A3)	12	12.63%
Slightly relevant (A4)	11	11.58%
Rather relevant (A5)	9	9.47%
Very relevant (A6)	4	4.21%
No answer	1	1.05%
Not completed or Not displayed	44	46.32%

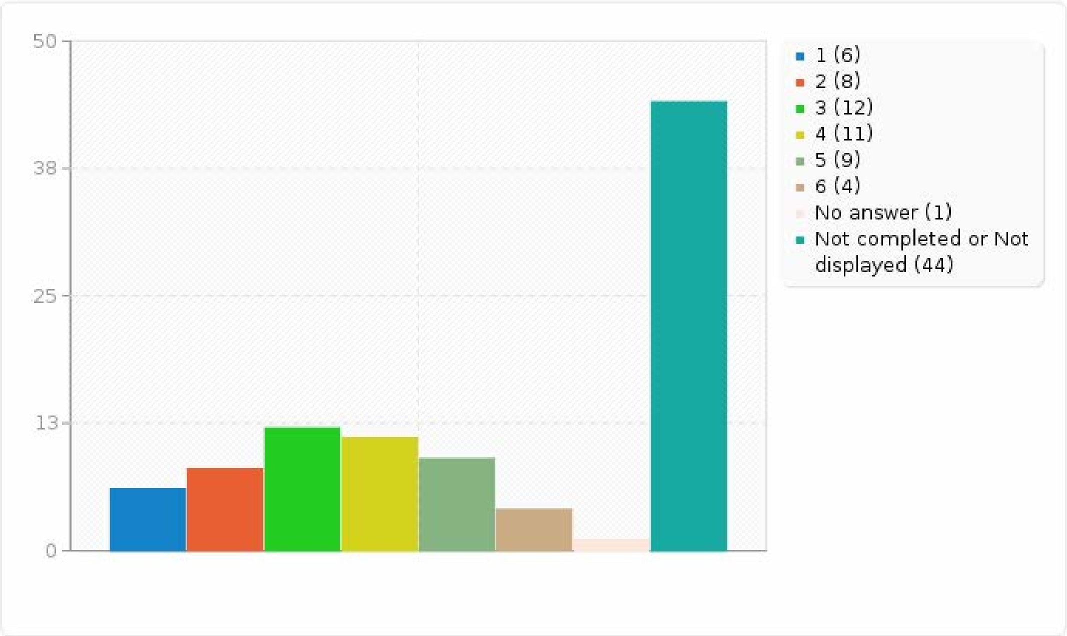

Field summary for Q16.4

Please rate to what extent the following aspects are important for the use of integrated protocols from the sponsor´s point of view.
[Number of amendments]

Answer	Count	Percentage
Not relevant at all (A1)	5	5.26%
Rather irrelevant (A2)	9	9.47%
Slightly irrelevant (A3)	14	14.74%
Slightly relevant (A4)	12	12.63%
Rather relevant (A5)	8	8.42%
Very relevant (A6)	2	2.11%
No answer	1	1.05%
Not completed or Not displayed	44	46.32%

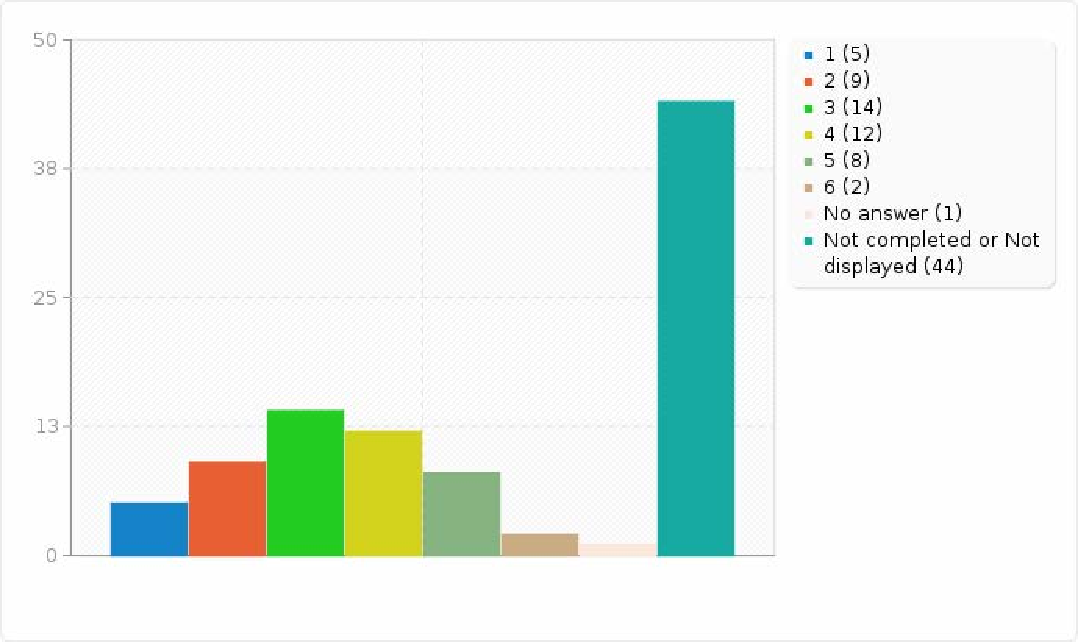

Field summary for Q16.5

Please rate to what extent the following aspects are important for the use of integrated protocols from the sponsor´s point of view.
[Expenditure for preparing clinical trial]

Answer	Count	Percentage
Not relevant at all (A1)	1	1.05%
Rather irrelevant (A2)	7	7.37%
Slightly irrelevant (A3)	14	14.74%
Slightly relevant (A4)	12	12.63%
Rather relevant (A5)	13	13.68%
Very relevant (A6)	3	3.16%
No answer	1	1.05%
Not completed or Not displayed	44	46.32%

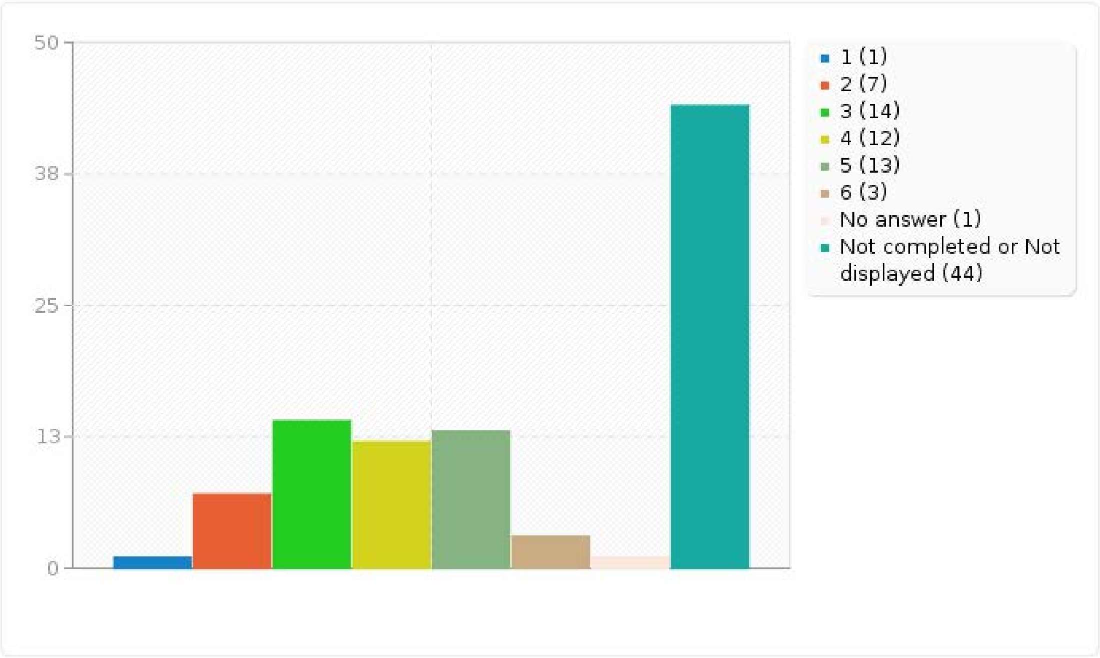

Field summary for Q16.6

Please rate to what extent the following aspects are important for the use of integrated protocols from the sponsor´s point of view.
[Expenditure for conducting clinical trial]

Answer	Count	Percentage
Not relevant at all (A1)	0	0.00%
Rather irrelevant (A2)	8	8.42%
Slightly irrelevant (A3)	11	11.58%
Slightly relevant (A4)	10	10.53%
Rather relevant (A5)	15	15.79%
Very relevant (A6)	6	6.32%
No answer	1	1.05%
Not completed or Not displayed	44	46.32%

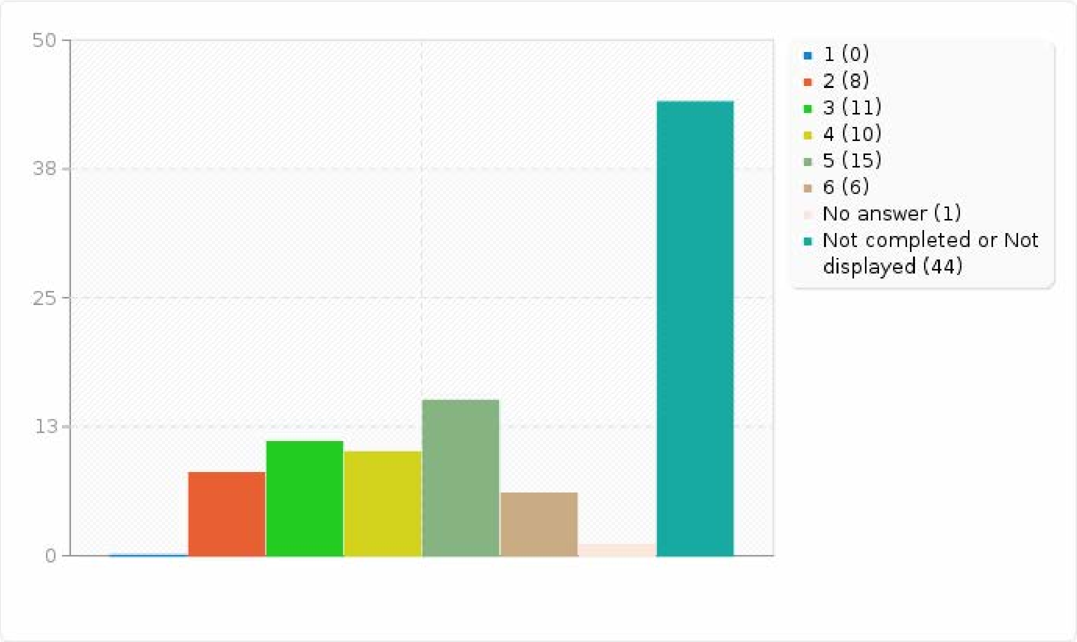

Field summary for Q17

On average, how many substantial amendments has your institution submitted per integrated phase 1 protocol?

Answer	Count	Percentage
none (A1)	9	9.47%
1 (A2)	11	11.58%
2 (A3)	15	15.79%
3 (A4)	10	10.53%
4 (A5)	3	3.16%
>=5, <=9 (A6)	2	2.11%
>=10 (A7)	0	0.00%
No answer	0	0.00%
Not completed or Not displayed	45	47.37%

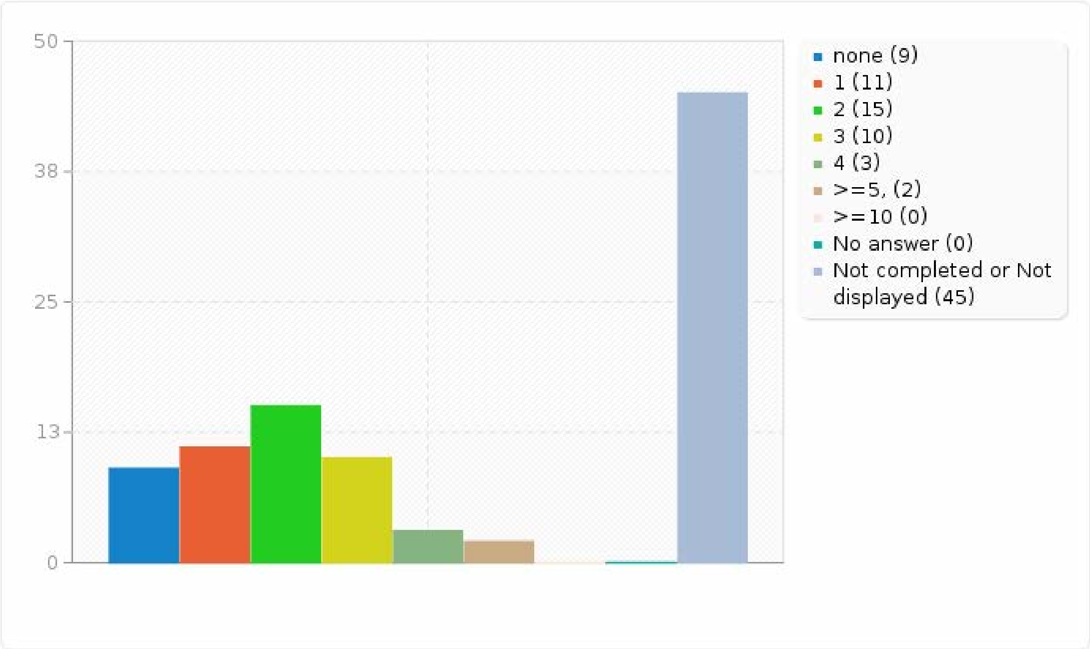

Field summary for Q18.1

Please rate, to what extent the following factors have resulted in time saved when using integrated protocols. [Avoidance of substantial amendments]

Answer	Count	Percentage
Clearly saved time (A1)	4	4.21%
Saved time (A2)	4	4.21%
Rather saved time (A3)	6	6.32%
Rather not time saved (A4)	5	5.26%
No time saved (A5)	7	7.37%
No time saved at all (A6)	6	6.32%
time delay (A7)	1	1.05%
No answer	17	17.89%
Not completed or Not displayed	45	47.37%

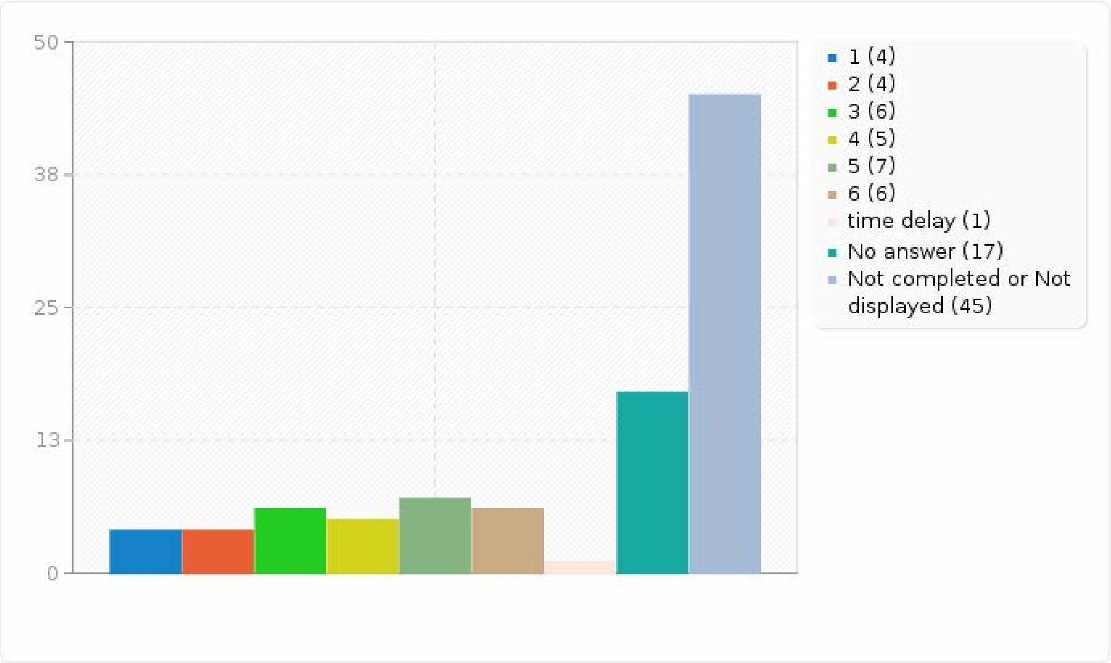

Field summary for Q18.2

Please rate, to what extent the following factors have resulted in time saved when using integrated protocols. [Avoidance of rejection of the clinical trial application (CTA) during initial submissions]

Answer	Count	Percentage
Clearly saved time (A1)	4	4.21%
Saved time (A2)	2	2.11%
Rather saved time (A3)	7	7.37%
Rather not time saved (A4)	2	2.11%
No time saved (A5)	3	3.16%
No time saved at all (A6)	11	11.58%
time delay (A7)	0	0.00%
No answer	21	22.11%
Not completed or Not displayed	45	47.37%

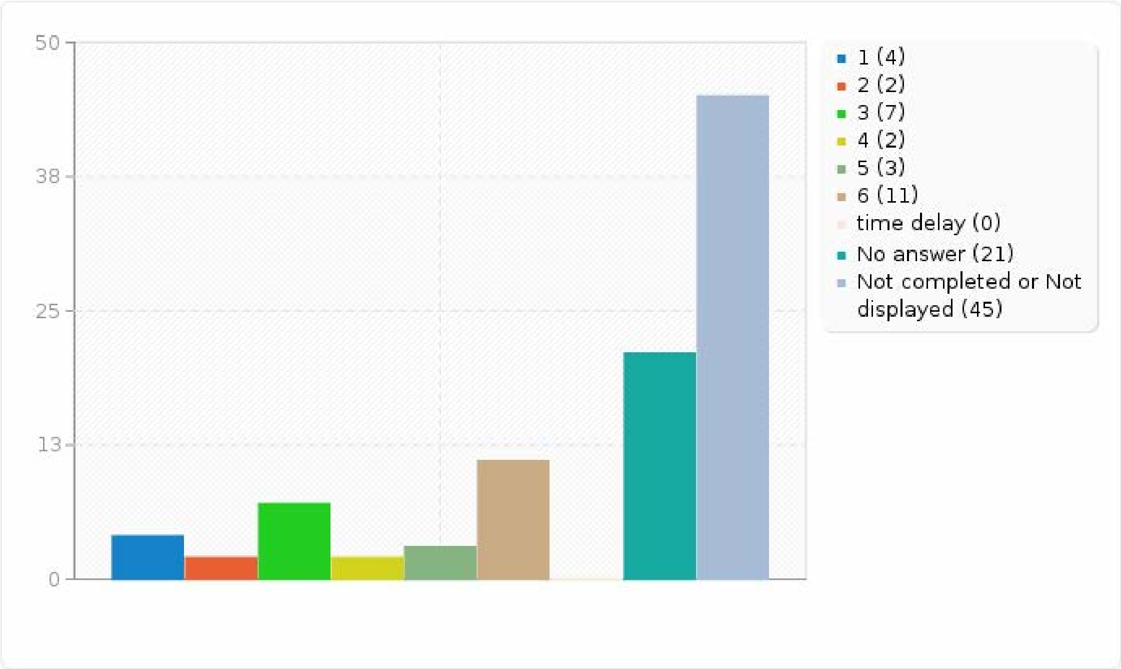

Field summary for Q18.3

Please rate, to what extent the following factors have resulted in time saved when using integrated protocols. [Time gained during initial submission to the BfArM]

Answer	Count	Percentage
Clearly saved time (A1)	8	8.42%
Saved time (A2)	4	4.21%
Rather saved time (A3)	4	4.21%
Rather not time saved (A4)	6	6.32%
No time saved (A5)	0	0.00%
No time saved at all (A6)	5	5.26%
time delay (A7)	1	1.05%
No answer	22	23.16%
Not completed or Not displayed	45	47.37%

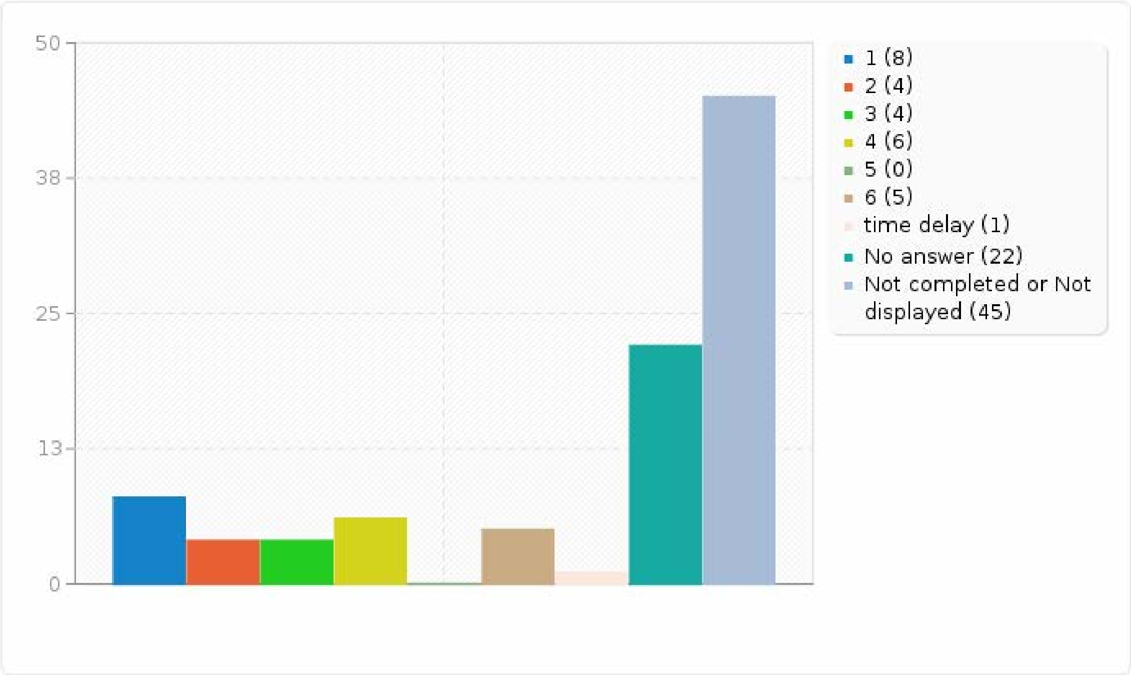

Field summary for Q19

Which response did your institution receive in the majority of cases when submitting an integrated protocol to your German competent federal higher authority (BfArM)?

Answer	Count	Percentage
Authorisation of clinical trial application (A1)	16	16.84%
Rejection of clinical trial application (A2)	1	1.05%
Objections/deficiency letter (A3)	16	16.84%
Other answers by participants (A4):	1	1.05%
1.) some minor changes go be incorporated		
No answer	16	16.84%
Not completed or Not displayed	45	47.37%

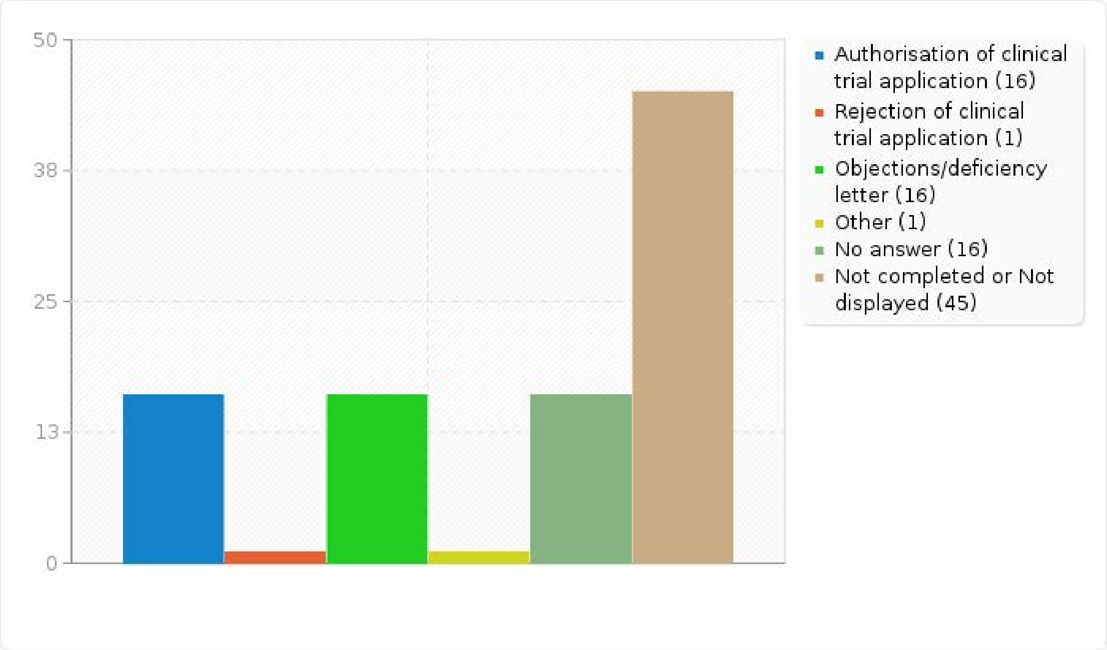

Field summary for Q20

Which were the main reasons for the rejection of an integrated protocol or for a deficiency letter by your German competent federal higher authority (BfArM)?

Answer	Count	Percentage
Not further specified (A1)	12	12.63%
Submission of interim results prior to starting a subsequent part of the clinical trial (A2)	19	20.00%
Inclusion/exclusion criteria for subjects/patients not/not sufficiently defined (A3)	10	10.53%
stopping rules (A4)	7	7.37%
Other answers by participants (A5):	10	10.53%

1.) not submitted any integrated protocol at the BfArM so far since BfArM is known to be critical in this regard. We go to other countries in which agencies are familiar with integrated protocols and getting an approval is no problem.

2.) dont know

3.) inappropriately large amount of questions on stability and purity of IP not received from HA in any other EU country

4.) not known to me

5.) usually accepted

6.) no rejections were given

7.) CMC, safety clarifications

8.) separate some of the sub-studies as considered with different objectives

9.) separate studies as consisered with different objectives

10.) insufficiently described trial parts

Not completed or Not displayed	45	47.37%

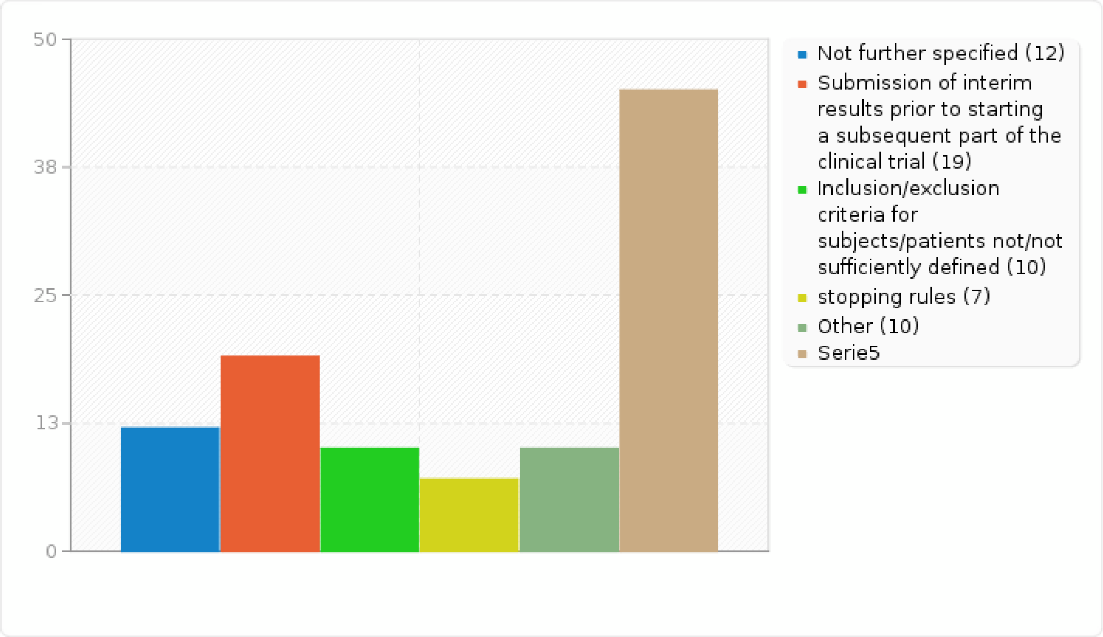

Field summary for Q21

Which of the following describes your institution most accurately?

Answer	Count	Percentage
Pharmaceutical Industry (A1)	43	45.26%
Contract Research Organisation (CRO) (A2)	3	3.16%
Academic Institution (e.g. University, non-profit research organisation) (A3)	3	3.16%
Other (A4):	0	0.00%
No answer	1	1.05%
Not completed or Not displayed	45	47.37%

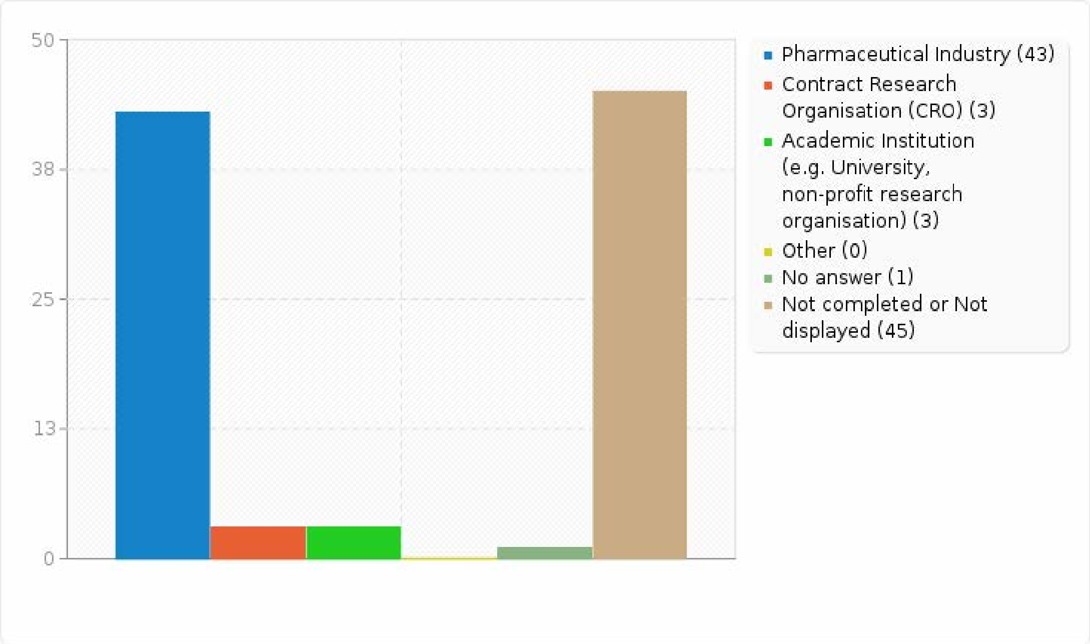
50
38
25
13
0
Pharmaceutical Industry (43)
Contract Research Organisation (CRO) (3)
Academic Institution (e.g. University, non-profit research organisation) (3)
Other (0)
No answer (1)
Not completed or Not displayed (45)
